# ROS generation and p-38 activation contribute to montmorillonite-induced corneal toxicity in vitro and in vivo

**DOI:** 10.1186/s12989-023-00519-9

**Published:** 2023-03-10

**Authors:** Jia Liu, Shubin Yang, Laien Zhao, Feng Jiang, Jianchao Sun, Shengjun Peng, Ruikang Zhao, Yanmei Huang, Xiaoxuan Fu, Rongrui Luo, Yu Jiang, Zelin Li, Nan Wang, Tengzheng Fang, Zhuhong Zhang

**Affiliations:** 1 School of Pharmacy, Key Laboratory of Molecular Pharmacology and Drug Evaluation (Yantai University), Ministry of Education, Collaborative Innovation Center of Advanced Drug Delivery System and Biotech Drugs in Universities of Shandong, Yantai University, Yantai, 264005 People’s Republic of China; 2 School of Chemistry and Chemical Engineering, Yantai University, Yantai, 264005 People’s Republic of China; 3 Department of Ophthalmology, Tianjin Medical University General Hospital, Tianjin, 300052 People’s Republic of China; 4 School of Environment and Material Engineering, Yantai University, Yantai, 264005 People’s Republic of China

**Keywords:** Corneal injury, Montmorillonite, Toxicity, Oxidative stress, p38 activation

## Abstract

**Background:**

Montmorillonite (Mt) and its derivatives are now widely used in industrial and biomedical fields. Therefore, safety assessments of these materials are critical to protect human health after exposure; however, studies on the ocular toxicity of Mt are lacking. In particular, varying physicochemical characteristics of Mt may greatly alter their toxicological potential. To explore the effects of such characteristics on the eyes, five types of Mt were investigated in vitro and in vivo for the first time, and their underlying mechanisms studied.

**Results:**

The different types of Mt caused cytotoxicity in human HCEC-B4G12 corneal cells based on analyses of ATP content, lactate dehydrogenase (LDH) leakage, cell morphology, and the distribution of Mt in cells. Among the five Mt types, Na-Mt exhibited the highest cytotoxicity. Notably, Na-Mt and chitosan-modified acidic Na-Mt (C-H-Na-Mt) induced ocular toxicity in vivo, as demonstrated by increases corneal injury area and the number of apoptotic cells. Na-Mt and C-H-Na-Mt also induced reactive oxygen species (ROS) generation in vitro and in vivo, as indicated by 2′,7′-dichlorofluorescin diacetate and dihydroethidium staining. In addition, Na-Mt activated the mitogen-activated protein kinase signaling pathway. The pretreatment of HCEC-B4G12 cells with N-acetylcysteine, an ROS scavenger, attenuated the Na-Mt-induced cytotoxicity and suppressed p38 activation, while inhibiting p38 activation with a p38-specific inhibitor decreased Na-Mt-induced cytotoxicity.

**Conclusions:**

The results indicate that Mt induces corneal toxicity in vitro and in vivo. The physicochemical properties of Mt greatly affect its toxicological potential. Furthermore, ROS generation and p38 activation contribute at least in part to Na-Mt-induced toxicity.

**Supplementary Information:**

The online version contains supplementary material available at 10.1186/s12989-023-00519-9.

## Introduction

The last few decades have undergone a revolution in the applied use of nanomaterials to medicine, industry, and pharmaceuticals. A variety of engineered nanomaterials, such as liposomes [1, 2], polymeric nanoparticles [3, 4], and inorganic nanomaterials [5, 6], have been investigated for their use in safe preservation and their controlled release for effective drug delivery. Among the current types of nanomaterials, nanoclay holds great promise for its enhanced therapeutic effects. In recent years, clay minerals and types of nanoclay have been intensively studied and are considered key materials due to their abundance, low costs, and environmentally friendly properties [7]. Clay minerals are essential components of many medicinal products such as Talc, Activated Attapulgite, Bentonite, and Magnesium Aluminum Silicate [8]. Clay minerals are also used in pharmaceuticals due to their biological activity. Among FDA approved drugs, clay minerals are mainly used to treat gastrointestinal and topical diseases [9]. Besides their applications in medicine and pharmaceuticals, because of their unique physicochemical properties, nanoclays, including montmorillonite (Mt), are being increasingly applied to a broad spectrum of industrial purposes, such as food packaging [10, 11], and wastewater treatment [12–14]. Yet this rapid expansion in the commercialization of Mt has raised human health concerns [15]. The potential for human exposure to these materials will inevitably increase; hence, a safety assessment of Mt is imperative to protect both the environment and human health.

Mt is the name for class of widely distributed silicate clay minerals that have a 2:1 type layered structure [16]. For a three-layer structure of Mt, each crystal platelet, is composed of two silicon-oxygen tetrahedral sheets sandwiching an aluminum-oxide octahedral sheet. The interlayer cations adsorbed by Mt mainly include Na^+^ and Ca^2+^; hence these Mts are respectively known as Na-montmorillonite (Na-Mt) and Ca-montmorillonite (Ca-Mt) [17, 18]. Given its good compatibility, high adsorption, and strong cation exchange ability, Mt has been widely studied as a drug delivery carrier for modulating drug release [19]. Widespread applications in medicine have led to the production of Mt and its derivatives. For example, the cross-linked chitosan nanocomposite hydrogels can be obtained by introducing Mt into chitosan, which not only may serve as a drug sustained release carrier but also improves the drug loading capacity of chitosan [20]. Further, a chitosan-Mt hydrogel was able to recruit natural cells and promote calvarial healing without additional therapeutic drugs or stem cells, suggesting it harbors tissue engineering potential [21]. A novel nanocomposite, this prepared by Mt as the matrix carrier, with Fe_3_O_4_ nanoparticles as filler, and carrageenan as stabilizer, was recently considered to play a role as a biologically active pH-responsive drug [22]. Notably, Mt also has been studied as a drug delivery carrier for the eye, to improve the bioavailability and efficacy of drugs. On the one hand, Mt is directly used as a drug delivery carrier, for example, 0.12 μmol of brimonidine (BMD) drug can be inserted into the interlayer of 1 g of Mt [23]. On the other hand, drugs can be inserted into the modified Mt and even further encapsulated as liposomes, to better regulate a drug’s release [19, 24–26].

The toxicity of Mt has received less attention. Applying a 0.05% concentration of Mt resulted in toxicity after its exposure to human embryonic kidney (HEK) cells and SiHa cervical cancer cells for 24 h; that study also indicated Mt-induced toxicity may be related to the size, aspect ratio, and concentration of Mt [15]. Research using the HepG2 hepatoma cell line has shown that Na-Mt has potential genotoxicity: Na-Mt can induce micronuclei formation under non-cytotoxic concentrations, thus potentially posing a risk to human health, especially under long-term exposure [27]. Nanoclays, which are separated from the clay portion of the soil, are currently used as adsorbents for the treatment of wastewater or hazardous leaks, or as media for oil well drilling, paints, and cosmetics [28]. Globally, 25,000 to 51,000 tons of nanoclays are used annually, including bentonite and organic modified clays, and there is a trend towards further increase [29]. Due to the extensive use of Mt composite materials and their common contact with the respiratory tract of various human populations, studies on the toxicity of unmodified and modified Mt in human lung cells and lung tissues of rats have been conducted [28, 29]. Both the original Mt and modified Mt led to toxicity in lung cells and lung tissues, which could cause their morphological changes to cells; however, the modifying Mt could change its main physical and chemical properties, including morphology and surface area, which changes the toxicity of lung cells and lung tissues [28, 29]. Nevertheless, the toxicity of Mt in other organs, especially the eye, has yet to be studied to provide empirical support for the safe development of new clay nanocomposites.

**Scheme 1:**
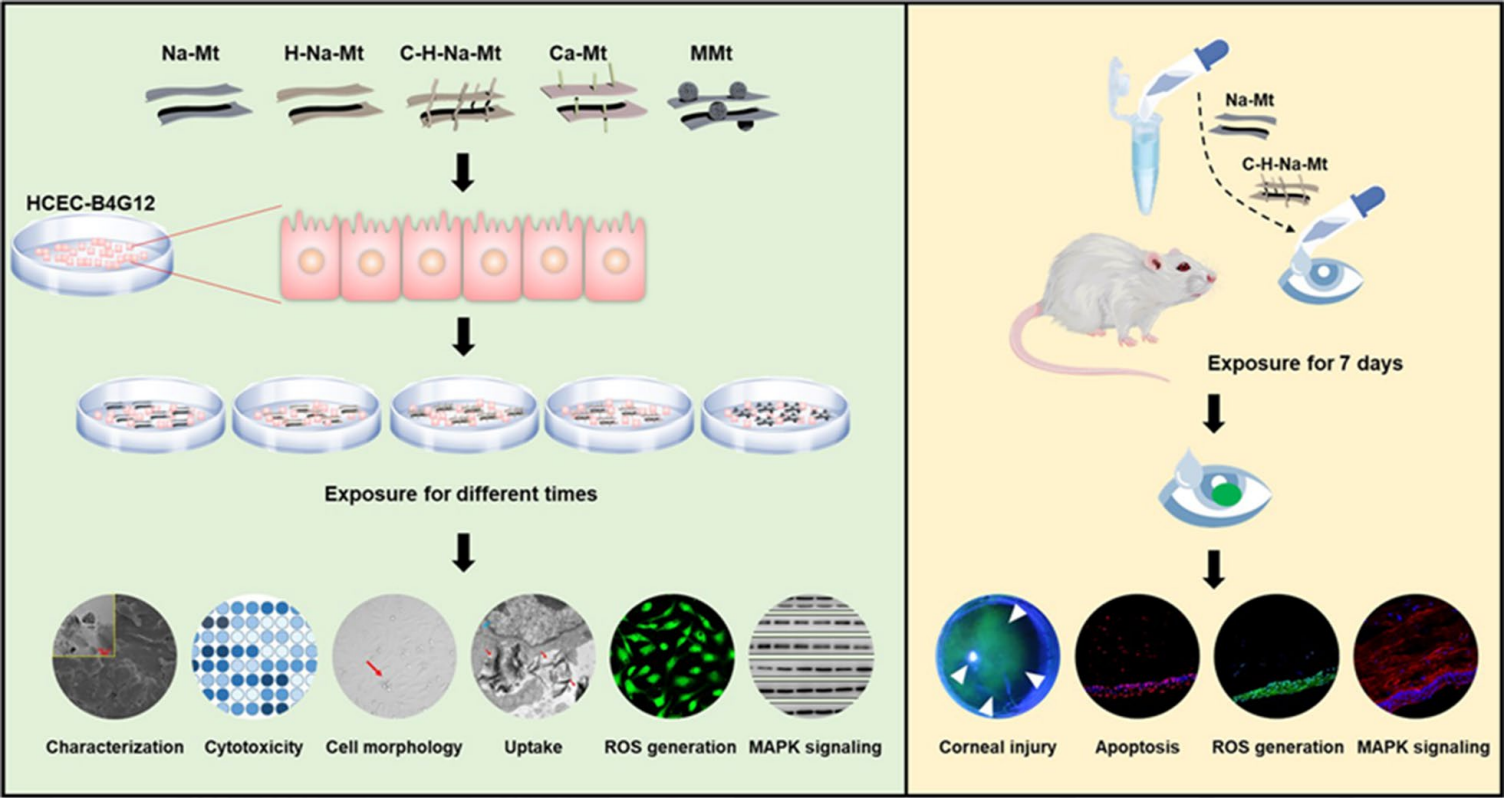
Schematic diagram of the experiments done in vitro and in vivo to evaluate the corneal toxicity induced by montmorillonite (Mt) with different properties. HCEC-B4G12 cells were treated with Na-Mt, H-Na-Mt, C-H-Na-Mt, Ca-Mt, and MMt to assess cytotoxicity, cell morphological changes, cell uptake, ROS production, and the expression of MAPK signaling proteins. Ocular irritation tests were performed on rats to monitor the area of corneal injury, corneal apoptosis, ROS generation in the cornea, and the expression of MAPK signaling proteins in the cornea of rats. Both in vitro and in vivo, the Mt types showed distinct corneal toxicity

In this study, we investigated the effects of different properties of Mt on corneal toxicity, both in vitro and in vivo. The Mt used here consisted of primitive Na-montmorillonite (Na-Mt) and Ca-montmorillonite (Ca-Mt), as well as prepared acidified Na-montmorillonite (H-Na-Mt), chitosan modified acidified Na-montmorillonite (C-H-Na-Mt), and magnetic montmorillonite (MMt) based on Na-Mt. Among them, Na-Mt, H-Na-Mt, and C-H-Na-Mt are used in some ocular drug delivery systems, including Brimonidine–montmorillonite hybrid formulation (BMD–MMT) [23], Montmorillonite/betaxolol hydrochloride complex (MT/BH complex) [19], and BH-Montmorillonite/chitosan nanoparticles (BH-Mt/CS NPs) [26], while Ca-Mt and MMt are used in water pollution treatment. All five Mt types were thoroughly characterized by analytical methods to determine their structure. This characterization revealed that the C-H-Na-Mt contains high C and N elements, and chitosan was successfully modified to the surface of H-Na-Mt. We hypothesized that chitosan may critically affect the corneal toxicity potential of Mt. It was revealed that chitosan indeed plays a pivotal role, in that chitosan functionalization of Mt mitigated its cytotoxicity. Additionally, we determined the roles of ROS and the MAPK signaling pathway in the toxicity from Mt. Since Mt as a drug carrier can prolong the residence time of the drug on the ocular surface, the eye, a sensitive organ, carries a high risk of Mt accumulation. To sum up, this is the first study of corneal toxicity and its mechanisms for five Mts with different properties conducted in vivo and in vitro, by addressing cytotoxicity, changed cell morphology, cell uptake of Mt, ROS production, oxidative stress, and signaling pathways (Scheme 1).

## Materials and methods

### Chemicals and reagents

Commercially available Na-montmorillonite was used (purity 97%; Zhejiang Sanding Group Co. Ltd., China). Sulfuric acid and other chemicals were purchased from Sinopharm Chemical Reagent Co., Ltd. All reagents used in this preparation of Mts are of analytical grade purity and were used without any further purification. Dulbecco’s modified eagle medium (DMEM), penicillin/streptomycin, and fetal bovine serum (FBS) were obtained from Life Technologies (Carlsbad, CA, USA). The 2′,7′-dichlorofluorescin diacetate (H_2_DCF-DA), N-acetylcysteine (NAC), p38 MAPK inhibitor, extracellular signal-regulated kinases (ERK) inhibitor and c-Jun N-terminal kinases (JNK) inhibitor were bought from Sigma-Aldrich (St. Louis, MO, USA). Dihydroethidium (DHE) came from the Beyotime Biotechnology Co., Ltd. (Shanghai, China), while fluorescein sodium was obtained from Macklin Biochemical Co., Ltd (Shanghai, China).

### Preparation of Mt with different properties

To prepare the H-Na-Mt, Na-Mt powder and a 3 mol/L H_2_SO_4_ solution were placed in a flat-bottomed flask and stirred at 80 °C for 4 h. After their reaction, each sample was centrifuged and washed with deionized water. After drying them at 80 °C for 12 h, the samples were ground and screened.

To obtain C-H-Na-Mt, firstly, 200 mg chitosan was dissolved with 40 mL of 5% acetic acid solution and stirred at 80 °C to obtain a 5 g/L chitosan stock solution. Secondly, 0.25 g of H-Na-Mt powder was placed in the plasma grafting reactor, and argon gas was injected into the reactor for 10 min at a pressure of 50 Pa with the power of 100 W. Thirdly, the treated H-Na-Mt was transferred to the prepared 80 mL 0.2 g/L chitosan solution (diluted from the 5 g/L chitosan stock solution) that was being heated at 80 °C, and continued to be heated and stirred at 80 °C for 24 h. C-H-Na-Mt composites were collected by centrifuging, washed several times with 5% acetic acid solution and endotoxin-free ultrapure water, and finally dried in a vacuum oven at room temperature. The MMt was synthesized via the hydrothermal method. To do this, 2.7 g of FeCl_3_·6H_2_O, 80 mL of ethylene glycol, 7.2 g of NaAC, and 2 g of sodium dodecyl sulfate (SDS) were added into the beaker and stirred magnetically for 0.5 h. Then 0.5 g of Mt was added and this stirred again for 0.5 h. The mixture was transferred to the PTFE inner tank for the hydrothermal reaction conducted at 180 °C for 10 h. Under the action of an external magnetic field, the solid and liquid are separated and the waste liquid from the upper layer is removed. The ensuing solids were transferred to a centrifugal tube, washed with ethanol and deionized water, and oven-dried at 60 °C.

### Cell culture and treatment with different types of Mt

HCEC-B4G12 (human corneal cell line) was obtained from Leibniz Institute DSMZ-German Collection of Microorganisms and Cell Cultures GmbH (DSMZ, Braunschweig, Germany) and cultured according to the recommended conditions. These cells were maintained in a complete medium, which consisted of 89% Dulbecco’s modified eagle medium, 10% FBS, and 1% penicillin/streptomycin. All cell cultures were incubated at 37 °C in a humidified environment with 5% CO_2_. Next, the five types of Mt (Na-Mt, H-Na-Mt, C-H-Na-Mt, Ca-Mt, and MMt) were respectively dispersed in phosphate-buffered saline (PBS) to prepare stock solutions (50 mg/mL). The stock solutions were diluted to different concentrations within a culture medium containing 2% FBS and 1% penicillin/streptomycin, just before the cells’ treatment. The cells were adjusted to a concentration of 1 × 10^5^ cells/mL, in a volume of 100 μL per well in 96- well plates, for the below toxicity assays.

### Cytotoxicity and morphological assessments

HCEC-B4G12 cells were collected and plated in 96-well plates at a density of 1 × 10^4^ cells/well and cultured overnight in a CO_2_ incubator. Next, the cells were exposed to Na-Mt, H-Na-Mt, C-H-Na-Mt, Ca-Mt, and MMt having different concentrations (1.56–100 μg/mL) for 24 and 48 h. As recently described [30], the ATP levels were measured by using the CellTiter-Lumi™ Plus Luminescent Cell Viability Assay (Beyotime, Beijing, China), while the LDH Release Assay (Beyotime, Beijing, China) was used to assess the membrane integrity of HCEC B4G12 cells, with cell viability examined by applying the Cell Titer 96 Aqueous One Solution Cell Proliferation Assay (Promega Corporation, Madison, WI, USA). Luminescence and absorbance were respectively recorded in a microplate reader (SpectraMax ID3, Molecular Devices, USA). Finally, morphological changes of HCEC-B4G12 cells were also examined under a phase-contrast microscope (Leica DM16000B, Germany).

### Cellular uptake

Using a dose of 50 μg/mL, the five types of Mt (Na-Mt, H-Na-Mt, C-H-Na-Mt, Ca-Mt, and MMt) were respectively used to treat HCEC-B4G12 cells for 24 h, after which the cells were seeded in 6-cm-diameter dish and cultured overnight at 37 °C in a 5%-CO_2_ incubator. The cells were washed three times with PBS and collected, then fixed overnight with 2.5% glutaraldehyde solution at 4 °C. After undergoing gradient dehydration and embedding, the sample cells were sectioned by ultrathin slicer, stained and dried, and examined with transmission electron microscope (TEM; Hitachi H7650, Japan).

### In vivo ocular irritation tests in rats

Healthy 3-week-old male Wistar rats were purchased from the Pengyue Experimental Animal Company (Jinan, China) and used in this study. Animal procedures conformed to the Association for Research in Vision and Ophthalmology Animal Statement and were approved by the Committee of Yantai University for the Care and Use of Laboratory Animals (Certification number: YTU20210901). All rats were placed in 12-h dark/light cycle under standard laboratory conditions, and allowed to get available water and food ad libitum. Before implementing the ocular irritation tests, the rats were randomly assigned to four groups: low dose Na-Mt, high dose Na-Mt, low C-H-Na-Mt, and high C-H-Na-Mt, and allowed to acclimate for 1 week. Next, 5 μL of Na-Mt (2 mg/mL and 10 mg/mL) or C-H-Na-Mt (2 mg/mL and 10 mg/mL) were dropped into the conjunctival sac of the left eye of rats, by gently pulling down the lower eyelid. Lids of rats were lightly closed for ca. 1 s to reduce any leakage of the Na-Mt and C-H-Na-Mt suspensions of different concentrations. The left eyes of rats in each group were exposed to differing concentrations of Na-Mt and C-H-Na-Mt three times per day at 6-h intervals for 1 week, while the right eye of each rat went untreated as the control.

### Corneal fluorescein staining

After 7 days of exposure to Mt as described in the above procedures, corneal injury and the extent (area) of this injury were assessed by a corneal fluorescein staining test. In brief, 3% fluorescein was dropped into the conjunctival sac of rats, and 2 min later the cornea of each rat was detected by cobalt blue light irradiation with slit lamp, and photographed. Further, visible signs of corneal edema and corneal opacification were looked for using a slit lamp microscope, and photographs also obtained.

### TUNEL assay

The One-step TUNEL Apoptosis Assay Kit (Beyotime, Beijing, China) was used to perform the TUNEL assay (terminal deoxynucleotidyl transferase-mediated dUTP nick end labeling assay) for 8-μm-thick corneal cryosections. Briefly, cryosections of the cornea samples were fixed with 4% paraformaldehyde for 60 min, and then washed thrice with PBS, followed by the addition of 0.3% Triton X-100 and incubation at room temperature for 5 min. Next, for the TUNEL staining, the TUNEL test solution was prepared according to the manufacturer’s instructions. To each sample, 50 μL of TUNEL solution was added and this incubated at 37 °C for 60 min in darkness. After rinsing three times with PBS for corneas, 4ʹ,6-diamidino-2-phenylindole (DAPI) was added to counterstain and seal each cryosection sample. Three cryosections from each rat’s cornea were analyzed and photographed by confocal laser scanning microscopy (CLSM; Zeiss LSM 800, Germany).

### Measurement of ROS in vitro and in vivo

The production of intracellular ROS in HCEC-B4G12 cells was detected by H_2_DCF-DA staining as described previously [30]. Briefly, H_2_DCF-DA (10 μM) was used to treat HCEC-B4G12 cells for 30 min in a cell culture incubator. To remove the unbound dye, the cells were washed three times with PBS and then exposed to Na-Mt and C-H-Na-Mt of various concentrations (3.13–50 μg/mL) with a phenol red-free medium. Subsequently, the cells were incubated continuously and the oxidation of H_2_DCF-DA was later detected by CLSM (Zeiss LSM 800, Germany) at 2, 6, 12, and 24 h. In vivo, the levels of corneal ROS were measured via DHE staining of 8-μm-thick corneal cryosections. Briefly, cryosections were fixed with 4% paraformaldehyde at room temperature for 60 min. Then, the sections in 10 μM DHE were incubated for 30 min in the dark, after which DAPI was added to counterstain and seal each after rinsing with PBS for corneas. Three sections of each rat were analyzed and photographed using CLSM (Zeiss LSM 800, Germany).

#### Immunofluorescence staining of the cornea

The eyeballs of rats were fixed with 4% paraformaldehyde for 2 h, then embedded with optimal cutting temperature compound (OCT) at –80 °C and cut into 8-μm-thick sections for their immunofluorescence staining. Cryosections were washed twice with PBS, permeabilized with 0.3% Triton X-100 in PBS, and blocked in 0.1% Triton X-100 and 2% bovine serum albumin (BSA) in PBS. The blocked corneal tissue was then incubated overnight at 4 °C with primary antibodies: these were p38, phospho-p38 MAPK (Thr180/Tyr182), ERK1/2, phospho-ERK1/2 (Thr202/Tyr204), JNK, and p-JNK (Thr183/Tyr185) (Cell Signaling Technology; Danvers, MA, USA). After the corneal cells were incubated with secondary antibodies, their nuclei were counterstained with DAPI. All sections were detected using CLSM (LSM 800, Zeiss, Germany).

#### Western blot analysis

Total cellular proteins were extracted from HCEC-B4G12 cells treated with Na-Mt, and protein concentrations determined by using the BCA Protein Assay Kit (Beyotime). The primary antibodies used for this were p38, phospho-p38 MAPK (Thr180/Tyr182), ERK1/2, phospho-ERK1/2 (Thr202/Tyr204), JNK, and p-JNK (Thr183/Tyr185) (Cell Signaling Technology; Danvers, MA, USA). Glyceraldehyde-3-phosphate dehydrogenase (GAPDH; Proteintech, 60,004-1-lg) served as the loading control for total cellular proteins. Western blot analysis was performed from three independent experiments for each treatment. To quantify the intensity of the resulting bands, Image J software was used (v1.53c).

#### Statistical analysis

To analyze the data, Graph Pad Prism 6 was used (Graph Pad Software; La Jolla, CA, USA), and results are expressed as the mean ± standard deviation (SD). For the experiments of NAC pretreatment and protein expression inhibition, statistical significance of their results was evaluated by one-way analysis of variance (ANOVA) followed by the Dunnett’s tests for the comparisons between different concentrations to the vehicle control, or two-way ANOVA followed by Sidak’s multiple comparisons test to distinguish which two treatment groups differed significantly. Differences between means were considered statistically significant when the resulting *p*-value was < 0.05.

## Results

### Characterization of Mt with different properties

**Fig. 1:**
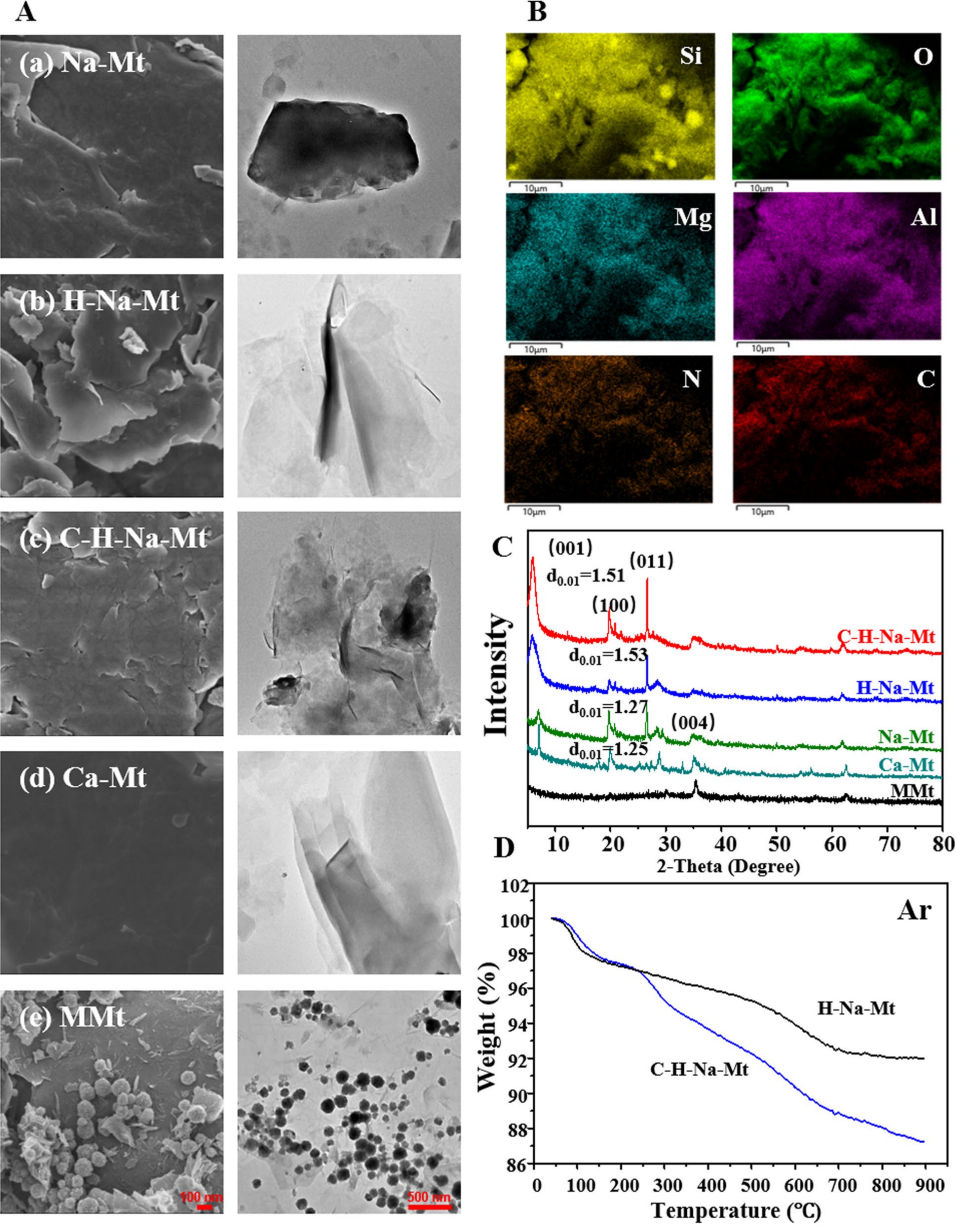
Characterization of montmorillonite (Mt) with different properties. **A** SEM (left panel) and TEM (right panel) images of Na-Mt (**a**), H-Na-Mt (**b**), C-H-Na-Mt (**c**), Ca-Mt (**d**), and MMt (**e**). **B** EDS mapping images of the C-H-Na-Mt composite. **C** X-ray diffraction peaks of Na-Mt, H-Na-Mt, C-H-Na-Mt, Ca-Mt, and MMt. **D** TGA curves of the H-Na-Mt and C-H-Na-Mt composites. Scale bars are 100nm (SEM) and 500 nm (TEM)

To better understand the differences between Mt and to verify that the preparation of modified Mt was successful, we first characterized the five types of Mt. The SEM and TEM micrographs of Na-Mt, H-Na-Mt, C-H-Na-Mt, Ca-Mt and MMt are shown in Fig. 1A. Na-Mt appears as a rock-like macroparticle with a rough, crumpled surface (Fig. 1a), this consistent with other research [13]. By way of comparison, significant differences were observed in Ca-Mt, which had the typical dense structure of Mt but a much smoother surface and far fewer cracks (Fig. 1d). After acid washing of Na-Mt, many wrinkles appeared at the edge of the flakes, resulting in disorderly stacked flakes and numerous uneven pores in H-Na-Mt composites (Fig. 1b). The montmorillonite structure was destroyed by H^+^ in sulfuric acid, during which time H^+^ replaced cations in the interlayer domain, resulting in a weakened interlayer bonding force of the Mt crystal layer and the peeling and reduction of lamellae and their acid dissolution, which is confirmed by the left-shift of the (001) reflection in XRD pattern (Fig. 1C). This result is also consistent with previous reports [31, 32]. Compared with H-Na-Mt, fewer nanosheets were found after the addition of chitosan (Fig. 1c), because some polymers were decorated on the surface of C-H-Na-Mt. Furthermore, C and N elements with a high mass fraction existed homogenously in the C-H-Na-Mt composites (Fig. 1B), indicating that chitosan was uniformly successfully modified to the surface of H-Na-Mt. In addition, the surface of MMt was also investigated. Plenty of spherical Fe_3_O_4_ nanoparticles (Fig. 1e) were observed in MMt.

As Fig. 1C shows, the pronounced characteristic peaks of Na-Mt at 2*θ* = 6.97°, 19.69°, and 28.41° correspond to the diffractions of (001), (100), and (004) crystal faces of montmorillonite (Beidellite PDF#43-0688). Especially, the (001) crystal face featured the largest diffraction intensity, being the main characteristic peak of Mt. The intense peaks seen at 2*θ* = 20.67° and 26.54° correspond to the diffractions of (100) and (011) crystal faces of quartz (PDF#70–3755) [31, 33]. According to the Bragg diffraction Eq. (2dsin*θ* = nλ) [34], the spacing distance (*d*) between the (001) planes of Na-Mt is ca. 1.27 nm. The XRD spectrum of Ca-Mt revealed a profile similar to Na-Mt. After the acid treatment, the *d*_*001*_ values increased to 1.53 nm, indicating a significantly exfoliated Na-Mt structure. Adding chitosan slightly changed the layer spacing due to surface modification, not an intercalation process. The main peaks of Fe_3_O_4_ (PDF#76–0956) were apparent in MMt [13], corresponding to Fe_3_O_4_ nanoparticles loaded onto MMt’s surface, which agrees with observations in Fig. 1A(e).

A thermogravimetric analysis (TGA) was done to study the thermodynamic stability of C-H-Na-Mt composites and to research the weight ratio of chitosan to Mt in this composite. In Fig. 1D are the TGA curves of the H-Na-Mt and C-H-Na-Mt composites under a flow of Ar atmosphere. The ca. 2.3% weight loss of H-Na-Mt up to 156 °C was related to the removal of water, with a negligible weight loss (~ 2.0%) from 156 to 450 °C. The second weight loss ratio (~ 3.6%), from 450 to 800 °C arose from self-dehydroxylation of the hydroxyl groups on the surface of H-Na-Mt [34]. For the C-H-Na-Mt composite, three clearly separated weight loss stages were evident: at 40–156 °C, 156–450 °C, and 450–800 °C, these mainly attributed to the combustion of absorbed water, the grafted chitosan, and the H-Na-Mt structure, respectively. Thus, the content of grafted chitosan was calculated as ~ 4.8% in the C-H-Na-Mt composite. For the characterization of the addition of chitosan, in addition to the TGA, XPS analysis and FTIR analysis were performed. The presence of Si, O, and N in the H-Na-Mt composite were observed (Additional file 1: Fig. S1), which is consistent with the EDS analysis in Fig. 1B. The typical N 1s peak (Additional file 1: Fig. S1A) is attributed to the –NH_2_ functional groups in chitosan, which is consistent with our previous study [34]. Compare with H-Na-Mt, obviously left shift in the position of XPS Si 2p peak and O 1s peak were observed in C-H-Na-Mt (Additional file 1: Fig. S1B & C), suggesting the deformation of silica tetrahedral sheet, and this result is consistent with our previous report [35]. Therefore, these findings suggest chitosan was grafted onto Mt. Due to the low concentration of chitosan in the C-H-Na-Mt composite (about 4.8 wt% from TGA analysis), the FTIR analysis of H-Na-Mt and C-H-Na-Mt did not show obvious difference.

### Cytotoxicity of different types of Mt in HCEC-B4G12 cells

**Fig. 2:**
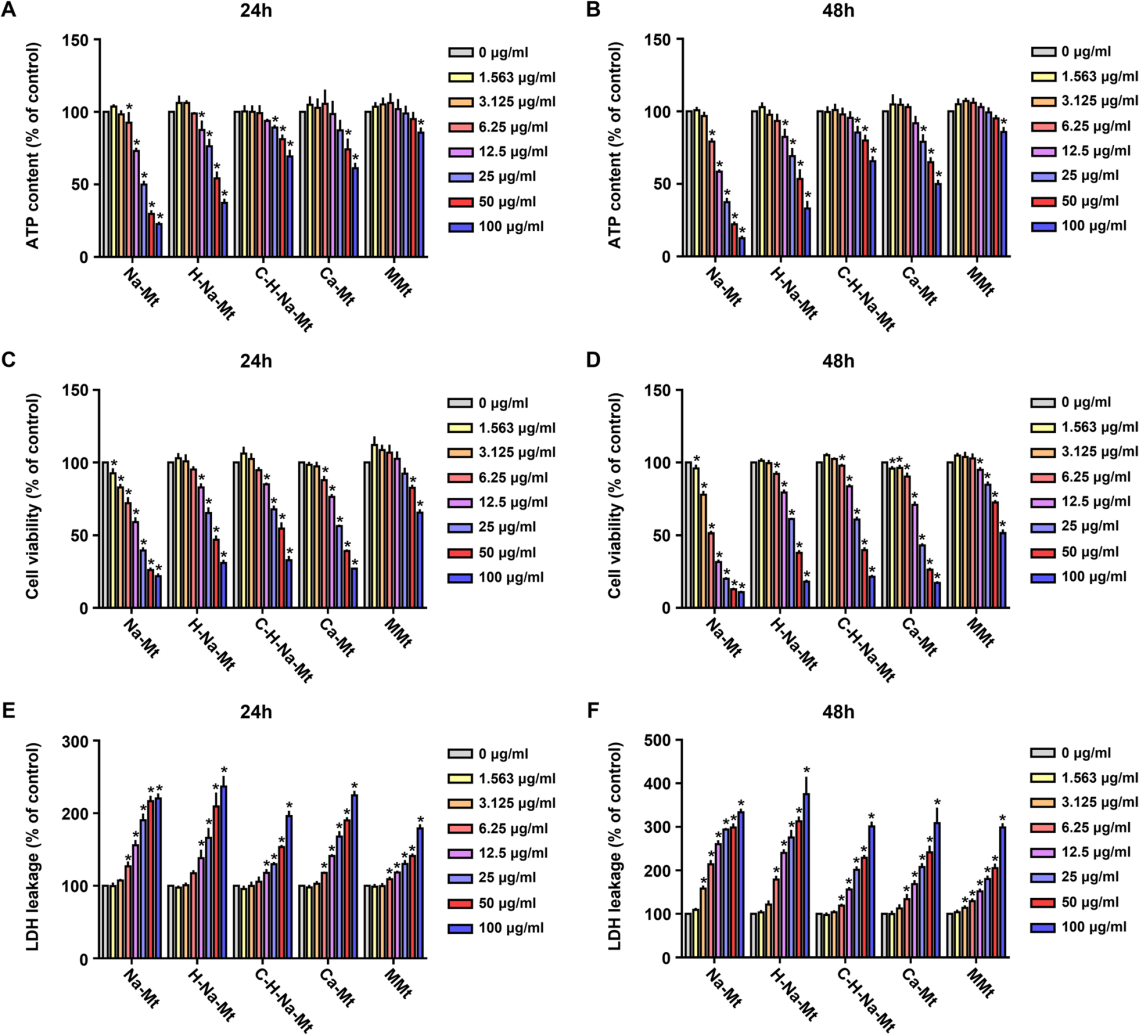
Cytotoxicity of montmorillonite (Mt) with different properties in HCEC-B4G12 cells. HCEC-B4G12 cells were treated with various concentrations (1.56–100 μg/mL) of five types of Mt for 24 h (**A**, **C** and **E**) and 48 h (**B**, **D** and **F**) before the measurements. **A**, **B** Effects on the ATP content of HCEC-B4G12 cells. **C**, **D** Cell viability examined using the CCK-8 assay. **E**, **F** Effects on membrane integrity of HCEC-B4G12 cells based on the LDH assay. Data points are the mean ± SD from three independent experiments with three parallel samples per concentration in each experiment. *p < 0.05 compared with the control

Mt reportedly poses potential genotoxicity to the HepG2 human hepatic cell line [27]. Here we investigated and compared the cytotoxicity of five types of Mt (Na-Mt, H-Na-Mt, C-H-Na-Mt, Ca-Mt, and MMt) in human HCEC-B4G12 corneal cells. It has been reported that FBS in the medium may affect the aggregation and toxicity of nanomaterials [36, 37]. To investigate the complete medium on the particle size of five kinds of Mt, we performed light microscopy (Additional file 1: Fig. S3A) and dynamic light scattering (DLS) (Additional file 1: Fig. S3B) to examine morphology and particle size after 48 h of incubation with complete medium containing different concentrations of FBS, including 2% and 10%. The result showed that the particle size of MMt and Ca-Mt decreased in an FBS concentration-dependent manner, while the particle size of other types of Mt did not change significantly (Additional file 1: Fig. S3). Therefore, to reduce the effect of FBS on toxicity and keep cells close to normal physiological state, we chose to use the medium with a 2% FBS concentration in Mt exposure. The in vitro Mt toxicity was assessed after 24 h and 48 h of treatment, by three parameters: ATP content, LDH leakage, and CCK8 viability assay (Fig. 2). Consistently, all Mt materials caused time- and concentration-dependent decreases in both the ATP content (Fig. 2A & B) and cell viability (Fig. 2C & D). Among them, Na-Mt generated the most severe cytotoxicity, followed by H-Na-Mt, being least for MMt. Compared with the control, the ATP content in HCEC-B4G12 cells treated with 100 μg/mL of Na-Mt for 48 h dropped to the level of approximately 12.6% (Fig. 2B). Nevertheless, only when the concentration reached 100 μg/mL did MMt significantly reduce the intracellular ATP content; the ATP content of HCEC-B4G12 cells exposed to 100 μg/mL of MMt for 48 h still remained at ca. 85.8% (Fig. 2B). The cell viability of HCEC-B4G12 exposed to five Mt types showed a similar trend to the treatments’ impact on intracellular ATP levels (Fig. 2C & D). For example, cell viability of HCEC-B4G12 cells treated with 100 μg/mL Na-Mt for 48 h dropped to 10.8%, while that of those cells exposed to 100 μg/mL MMt for 48 h could persist at a much higher level of 51.4% (Fig. 2D). Further, the magnitude of LDH leakage in cells reflects the effect of exposure to Mts upon cell membrane integrity. As shown in Fig. 2E, applying the Na-Mt treatment at a concentration of only 6.25 μg/mL nonetheless induced a significant release of LDH in HCEC-B4G12 cells within 24 h, with an 17.8% increase in LDH leakage compared to the control group. Note, however, that the LDH release of HCEC-B4G12 cells treated with 6.25 μg/mL MMt also showed a significant difference at 24 h, albeit increased by only 9.2% over the control group (Fig. 2E). Moreover, the level of MMt-induced intracellular release of LDH was the lowest (Fig. 2E & F). Altogether, different types of Mt caused varying degrees of cytotoxicity in a dose-dependent manner as evidenced by a decrease in ATP content, inhibition of cell viability and LDH release. Besides, LDH release at 48 h of Mt treatment was obvious higher than that at 24 h, suggesting that the destruction of cell membrane integrity by Mt would be exacerbated with time.

**Fig. 3:**
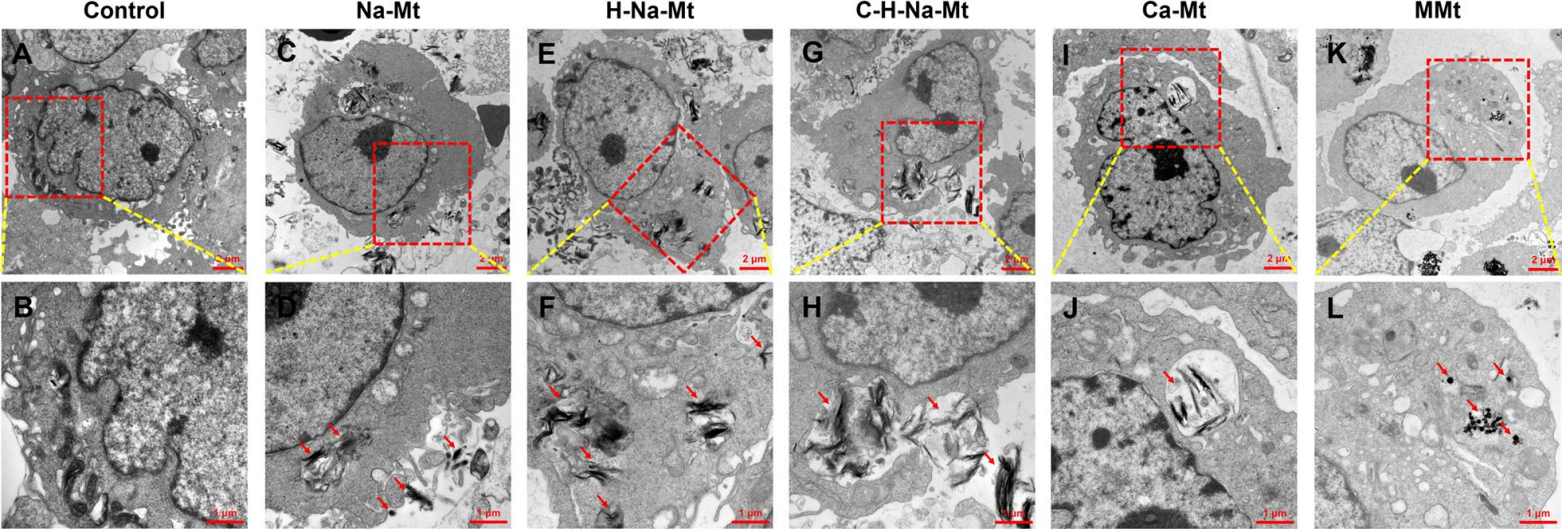
TEM assay of cellular uptake and localization of montmorillonite (Mt) with different properties in HCEC-B4G12 cells. HCEC-B4G12 cells were exposed to five types of Mt (50 μg/mL) for 24 h. **A**, **B** TEM images showing the ultrastructure of control HCEC-B4G12 cells. Consistent with (**A**, **B**) but for HCEC-B4G12 cells exposed to Na-Mt (**C**, **D**), H-Na-Mt (**E**, **F**), C-H-Na-Mt (**G**, **H**), Ca-Mt (**I**, **J**), and MMt (**K**, **L**). Red arrowheads indicate the localization of five types of Mt in HCEC-B4G12 cells. Scale bars are 2 μm (**A**, **C**, **E**, **G**, **I**, **K**) and 1 μm (**B**, **D**, **F**, **H**, **J**, **L**)

### Localization of different types of Mt in HCEC-B4G12 cells

Using TEM, the in vitro uptake and the intracellular distribution of Mt with different properties were analyzed for HCEC-B4G12 cells. Results are presented for representative TEM images of HCEC-B4G12 cells treated with Na-Mt (Fig. 3C & D), H-Na-Mt (Fig. 3E & F), C-H-Na-Mt (Fig. 3G & H), Ca-Mt (Fig. 3I & J), and MMt (Fig. 3K & L). In the control HCEC-B4G12 cells not exposed to Mt (Fig. 3A & B), we could not detect the distribution of any Mt type in either cell membrane or cytosol. After 24 h of exposure, the five Mt types were respectively visible in the cytoplasm, and some Na-Mt had even aggregated around the cell membrane. Compared with the control, the nuclear area of cells exposed to the five types of Mt was significantly reduced relative to their total cell area. These findings suggest that Mt-induced cytotoxicity may be due to their entry into cells rather than just their effects on the cell surface.

### Effects of different types of Mt on the morphology of HCEC-B4G12 cells

**Fig. 4:**
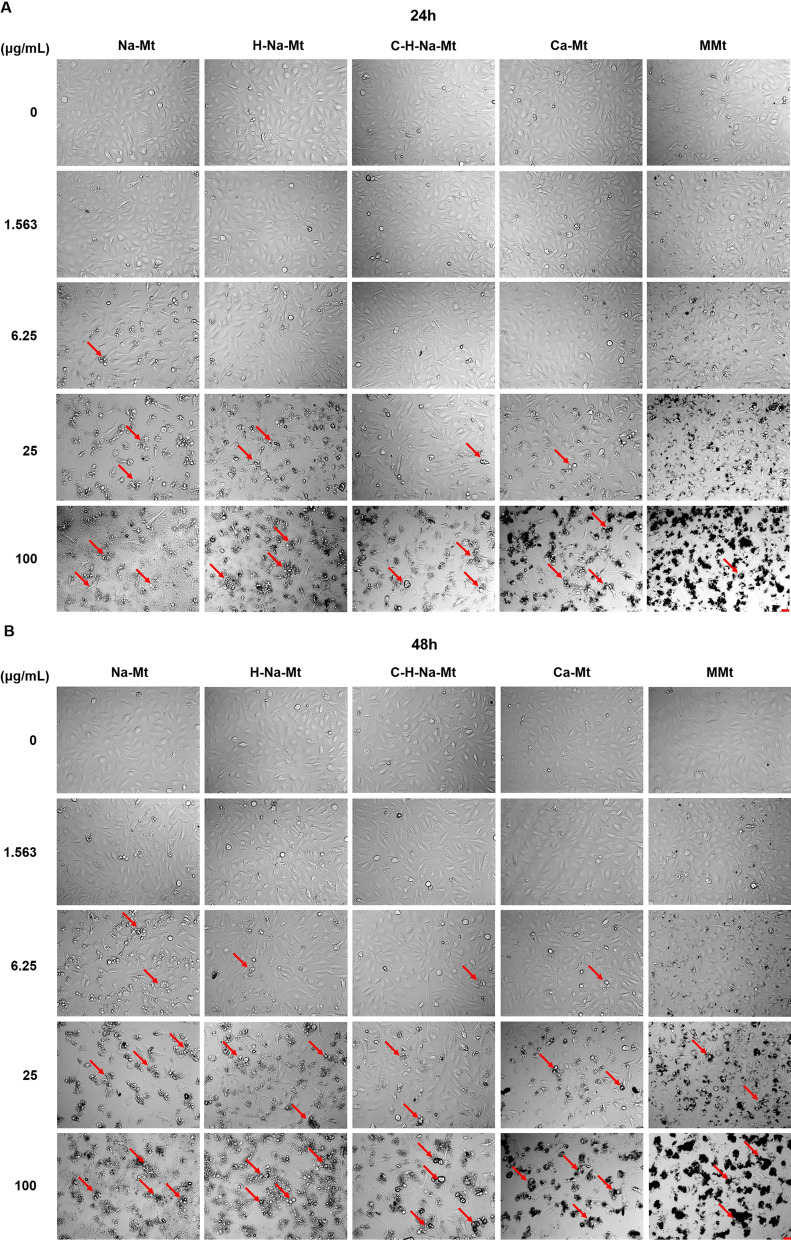
Morphological images of HCEC-B4G12 cells under a bright field view. Morphological changes in HCEC-B4G12 cells were observed by phase-contrast microscopy following 24 h (**A**) and 48 h (**B**) exposure to five types of montmorillonites with corresponding concentrations. Scale bar: 25 μm

The morphology of HCEC-B4G12 cells changed in response to an increasing concentration of Na-Mt, H-Na-Mt, C-H-Na-Mt, Ca-Mt, and MMt. At 24 h (Fig. 4A), the morphology of HCEC-B4G12 cells became irregular when exposed to 6.25 μg/mL of Na-Mt, 25 μg/mL of H-Na-Mt, C-H-Na-Mt, and Ca-Mt, and 100 μg/mL of MMt. At 48 h (Fig. 4B), the changes in cell morphology became more prominent with greater treatment concentrations. Under 100 μg/mL exposure to all five Mt types, most of the HCEC-B4G12 cells were detached, being round with cell debris, and their density evidently reduced. Consistent with cytotoxicity, Na-Mt impacted cell morphology the most, while MMt had the least effect.

### Mt caused corneal damage and apoptosis in vivo

**Fig. 5:**
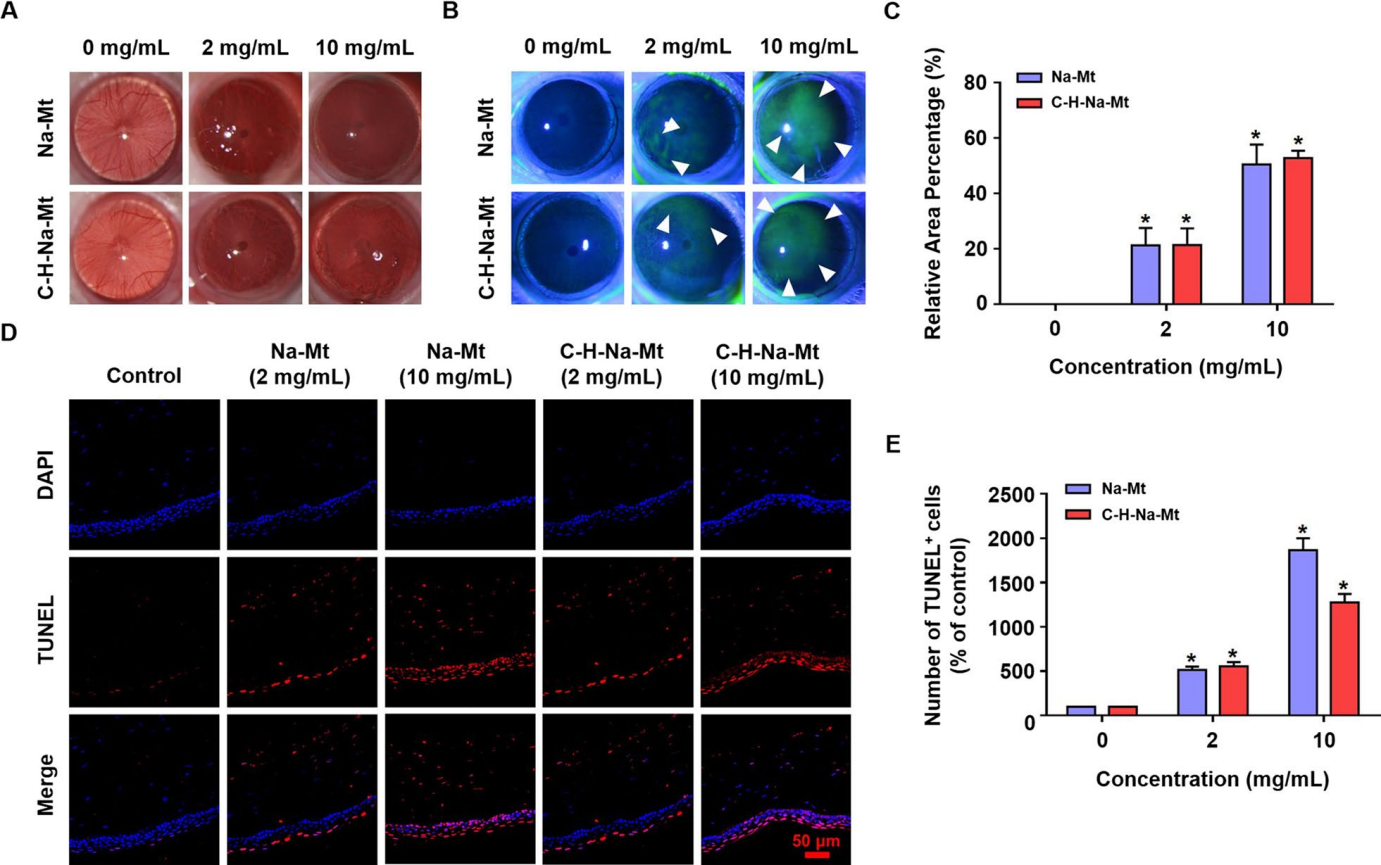
Corneal damage and apoptosis caused by montmorillonites (Mts) in vivo. **A** Representative photos of the anterior ocular segment of rat eyes treated with different concentrations (2 and 10 mg/mL) of Na-Mt or C-H-Na-Mt. **B** Representative images of corneal injury, under cobalt blue light, incurred after exposure to different concentrations (2 and 10 mg/mL) of Na-Mt or C-H-Na-Mt were detected by fluorescein sodium staining. The extent of corneal damage is indicated by the green fluorescence area. White arrowheads point to damage of the cornea. **C** Statistical analysis of the corneal damage area of green fluorescence at 7 days following exposure to different concentrations (2 and 10 mg/mL) of Na-Mt or C-H-Na-Mt. **D** Representative images of immunofluorescence of rat cornea for TUNEL (red) and DAPI (blue). The left eye of rats was dripped with Na-Mt or C-H-Na-Mt. At 7 days post-exposure, corneal sections were prepared for fluorescence microscopy for their analysis by the TUNEL assay. **E** The number of TUNEL^+^ cells of corneal sections treated with Na-Mt versus C-H-Na-Mt. Data are presented as the mean ± SD. *p < 0.05 compared with the control. Scale bars: 50 μm

According to the above in vitro experimental results, all five Mt types—Na-Mt, H-Na-Mt, C-H-Na-Mt, Ca-Mt, and MMt—showed toxicity in HCEC-B4G12 cells, of which Na-Mt was the most toxic and MMt the least toxic. In addition, Na-Mt, H-Na-Mt and C-H-Na-Mt have been studied in the treatment of glaucoma, such as BMD–MMT [23], MT/BH complex [19], BH-Mt/CS NPs [26]. Therefore, we selected Na-Mt and C-H-Na-Mt, two materials with relatively discernible toxicity and closely tied to medical applications to eyes, as representatives for in vivo experimentation. Based on the existing studies on the use of Mt as an ocular drug delivery carrier, which used a sample concentration of 2.8 mg/mL to drop into the lower conjunctival sac of each cornea for eye-irritation test in vivo [19, 25, 26], we chose 2 mg/mL as the starting dose to be tested in the in vivo corneal toxicity study. Furthermore, According to the Organization for Economic Co-operation and Development (OECD) Guidelines for the Testing of Chemicals, Test Guideline No. 405 In Vivo Eye Irritation/Serious Eye Damage in the OECD toxicity test standard [38], the maximum dose to the eye should not exceed 0.1 mL in volume or 100 mg in weight. Since this in vivo study only set two groups, so we set the high-dose group to be fivefold that of the low-dose group, that is, 10 mg/mL, and the administration volume was 5 µL. Therefore, we investigated the in vivo corneal toxicity of Na-Mt and C-H-Na-Mt at concentrations of 2 mg/mL (low dose group) and 10 mg/mL (high dose group). To assess the toxic effects of Mt in vivo, Na-Mt or C-H-Na-Mt was repeatedly exposed to the corneal surface of rats for 1 week, followed by corneal imaging and TUNEL assay performed using the slit lamp microscope and CLSM, respectively. As shown in Fig. 5, compare with the control, when the eyes of rats were exposed to Na-Mt or C-H-Na-Mt their cornea became rough and cloudy (Fig. 5A), with a large extent of damaged areas corresponding to the green patchy staining (Fig. 5B), and many apoptotic cells appearing in corneal tissue (Fig. 5D). As shown in Fig. 5C, at 1-week post-exposure, Na-Mt and C-H-Na-Mt caused a similar range of corneal damage, with a ca. 20% and 50% increase relative to the control in the cornea- injured area of rats exposed to a low dose (2 mg/mL of Na-Mt and C-H-Na-Mt) and high-dose groups (10 mg/mL of Na-Mt and C-H-Na-Mt), respectively. As evinced by Fig. 5E, compared with the control eye of rats, the exposure to 2 mg/mL of Na-Mt or C-H-Na-Mt increased the number of TUNEL-positive cells by ca. fivefold, whereas exposure to 10 mg/mL Na-Mt and C-H-Na-Mt increased them ca. 19- or 13-fold, respectively. Taken together, these results revealed greater a damaged area and abundance of apoptotic cells with higher concentrations of Na-Mt and C-H-Na-Mt. Moreover, a high dose of Na-Mt induced more apoptotic cells in vivo than did C-H-Na-Mt.

### Mt induced ROS overproduction in vitro and in vivo

**Fig. 6:**
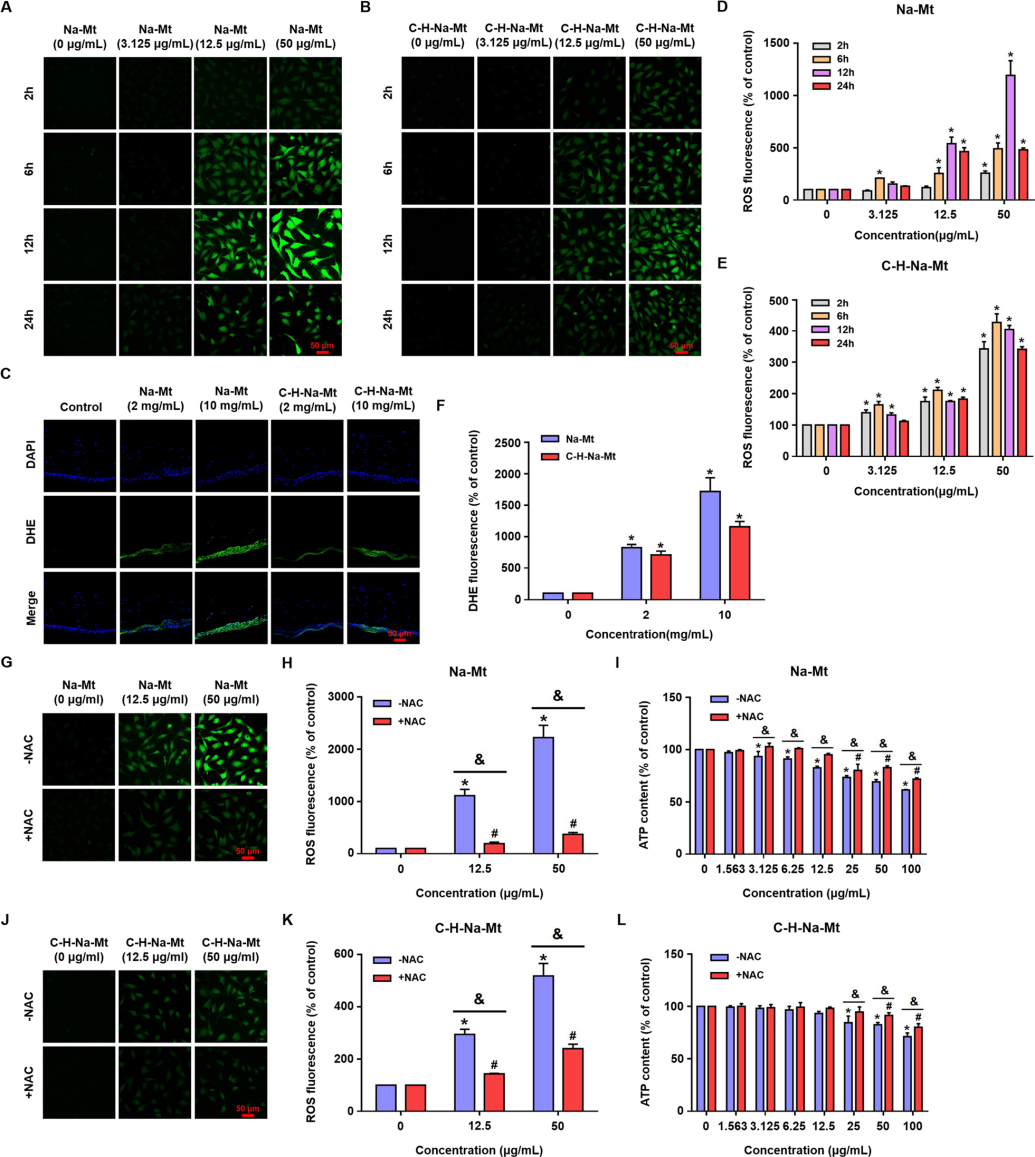
ROS overproduction induced by montmorillonites (Mts) in vitro and in vivo, and the effects of NAC pretreatment on Mt-induced cytotoxicity in vitro. **A**, **B** Under CLSM, ROS levels of HCEC-B4G12 in vitro were investigated by H_2_DCF-DA staining (green) at 2, 6, 12 and 24 h after exposure to Na-Mt (**A**) and C-H-Na-Mt (**B**) applied in various concentrations (0, 3.125, 12.5, and 50 μg/mL). **D, E** Quantification of the ROS fluorescence intensity in (**A**, **B**). **C** Representative DHE staining (green) of the corneal section of rats after their exposure to different concentrations (2 and 10 mg/mL) of Na-Mt or C-H-Na-Mt for 7 days in vivo. Nuclei were stained by DAPI (blue). **F** Quantification of ROS fluorescence intensity of different groups in (**C**). Data are presented as the mean ± SD. * p < 0.05 compared with the control. **G**, **J** Representative images of ROS levels of HCEC-B4G12 monitored by CLSM. HCEC-B4G12 cells were pretreated with 10 mM NAC for 1 h before exposing them to different concentrations (12.5 and 50 μg/mL) of Na-Mt or C-H-Na-Mt for additional 6 h. **H**, **K** Quantification of ROS fluorescence intensity in (**G**, **J**). **I**, **L** ATP content was measured at 12 h post-exposure to Na-Mt and C-H-Na-Mt, with and without pretreatment with NAC. Data points are the mean ± SD from three independent experiments, with three parallel samples per concentration in each experiment. *p < 0.05 compared with the control lacking the NAC pretreatment. ^***#***^*p* < 0.05 compared with the control receiving the NAC pretreatment. ^*&*^*p* < 0.05 between treatments with and without NAC pretreatment at the same concentration of different Mt. Scale bars: 50 μm

To gain mechanistic insight into Mt-induced toxicity effects, we tested whether Mt induced ROS overproduction in vitro and in vivo. HCEC-B4G12 cells were treated with Na-Mt (3.125, 12.5, and 50 μg/mL) and C-H-Na-Mt (3.125, 12.5, and 50 μg/mL), and their ROS production monitored at 2, 6, 12, and 24 h. ROS generation occurred as early as the 2-h time-point under the 50 μg/mL Na-Mt treatment (Fig. 6A). Maximum ROS induction was 12-fold that of the vehicle control at 12 h and 50 μg/mL Na-Mt treatment. At 24 h, although ROS generation was still significantly high in comparison with the control, the levels were less prominent than those at earlier time-points (Fig. 6A & D). Reduced ROS levels may be due in part to cellular antioxidant response mechanisms and reduced cell viability. The accumulation of ROS in the C-H-Na-Mt treatment group showed a similar trend to that in the Na-Mt treatment group (Fig. 6B & E). For example, ROS induction was detected as early as the 2 h mark under the 3.125 μg/mL C-H-Na-Mt treatment. Maximum ROS induction was fourfold that of the vehicle control at 6 h under the 50 μg/mL Na-Mt treatment.

To investigate the accumulation of Mt-induced ROS in vivo, we directly exposed the ocular surface of Wistar rats to Na-Mt or C-H-Na-Mt (2 and 10 mg/mL). Consistent with in vitro results, ensuing ROS production was also concentration-dependent (Fig. 6C). Exposure to Na-Mt and C-H-Na-Mt at different concentrations increased ROS levels in rat corneal tissue when compared with the untreated control. Compared with the vehicle control, ROS levels increased by 8- and 17-fold at 7 days after exposure to 2 and 10 mg/mL Na-Mt, respectively (Fig. 6F); the corresponding increases for C-H-Na-Mt exposure were 7- and 12-fold (Fig. 6F). Hence, the Na-Mt treatment induced higher levels of ROS production than did C-H-Na-Mt treatment in vivo. The augmented ROS levels suggested Na-Mt and C-H-Na-Mt caused oxidative stress.

### NAC pretreatment attenuated Mt-induced cytotoxicity

To further investigate the role of ROS production in the cytotoxicity of Mts, we used NAC (a ROS scavenger) to inhibit the levels of intracellular ROS. As shown in Fig. 6G & H & J & K, HCEC-B4G12 cells were pretreated with 10 mM NAC for 1 h prior to their exposure to 12.5 and 50 μg/mL of Na-Mt or C-H-Na-Mt for 6 h. The images of CLSM based on ROS fluorescence staining indicated that the NAC pretreatment of cells significantly suppressed ROS generation. Further, pretreating the HCEC-B4G12 cells with NAC (10 mM) for 1 h, prior to their treatment with 1.56 − 100 μg/mL Na-Mt and C-H-Na-Mt for 12 h alleviated Mt-induced cytotoxicity, as evidenced by the reductions in ATP content (Fig. 6I & L) These results revealed that the cytotoxicity of types of Mt was partly mediated by ROS generation.

### Mt activates the MAPK signaling pathway in vitro and in vivo

**Fig. 7:**
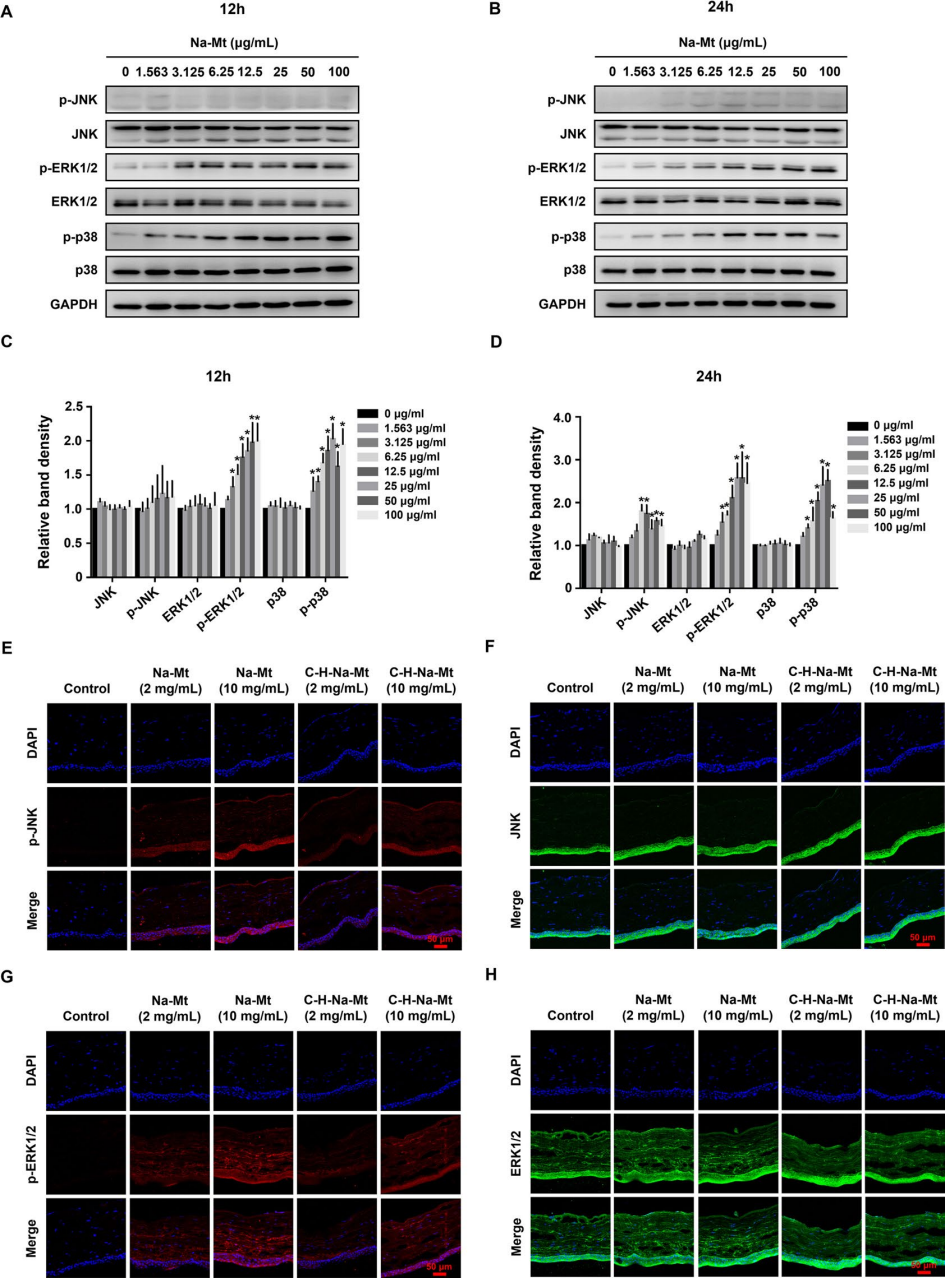

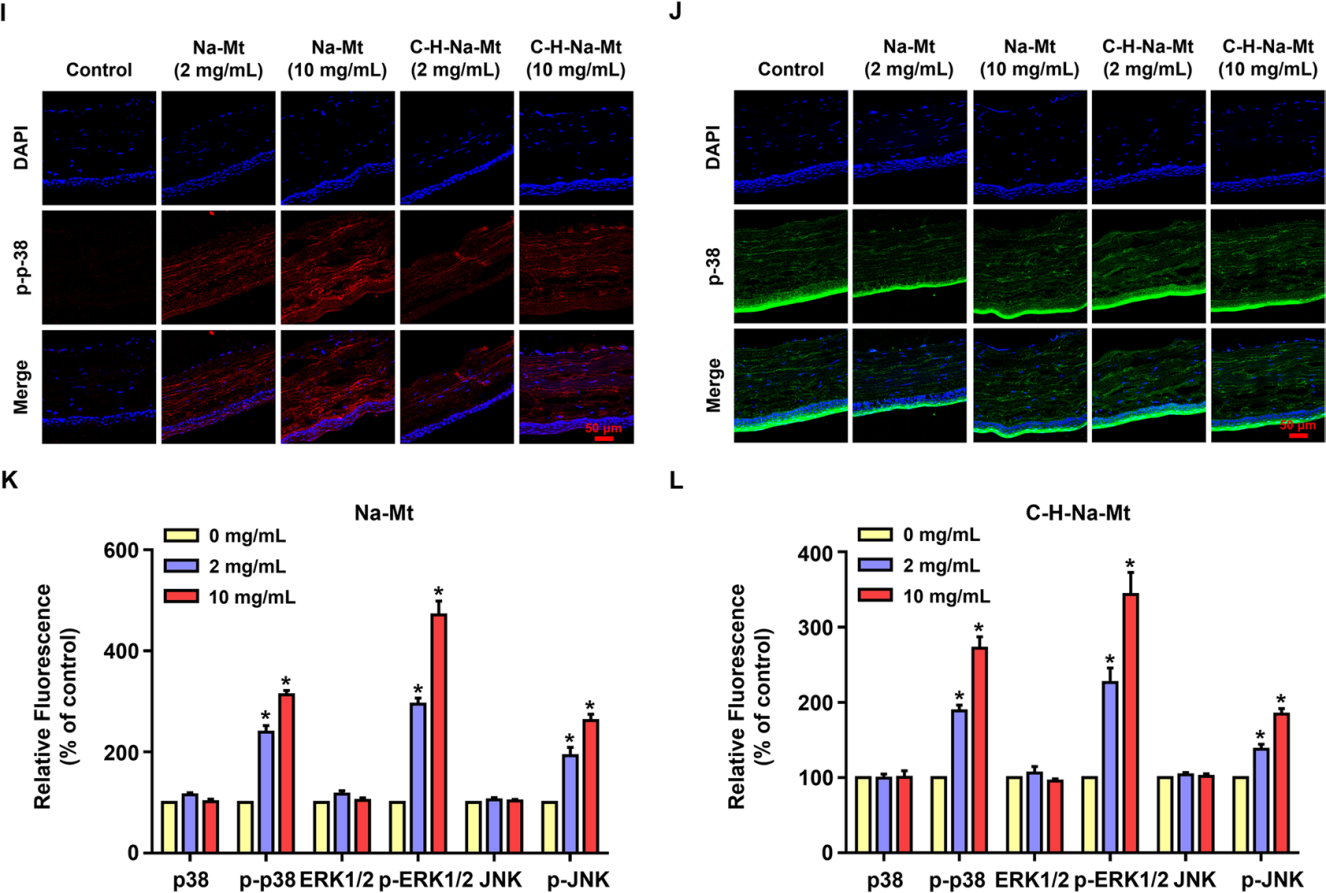
Effects of montmorillonite (Mt) on the MAPK signaling pathway in vitro and in vivo. **A**, **B** Representative bands of the western blot of JNK, p-JNK, ERK1/2, p-ERK1/2, p38, and p-p38 in the total proteins. Total proteins were extracted from HCEC-B4G12 cells exposed to various concentrations (1.56–100 μg/mL) of Na-Mt for 12 h and 24 h. **C**, **D** Quantification of JNK, p-JNK, ERK1/2, p-ERK1/2, p38, and p-p38 in **A**, **B** according to the densitometric analysis. Intensities of bands were normalized to the amount of GAPDH. *p < 0.05 between the Na-Mt treatment and the control. **E**–**J** Representative images indicating the expression of p-JNK (**E**, red), JNK (**F**, green), p-ERK1/2 (**G**, red), ERK1/2 (**H**, green), p-p38 (**I**, red), and p38 (**J**, green), in the cornea. Corneal sections were prepared from rats after exposing them to different concentrations (2 and 10 mg/mL) of Na-Mt or C-H-Na-Mt for 7 days. Nuclei were stained by DAPI (blue). (**K**, **L**) Quantified fluorescence intensity of the proteins in (**E**–**J**). *p < 0.05 compared with the control. Scale bars: 50 μm

JNK, ERK1/2, and p38 MAPK, three important members of mitogen-activated protein kinase (MAPK) family, are closely associated with apoptosis and oxidative stress [39, 40]. To determine whether Mt triggers MAPK signaling, the expression and phosphorylation of MAPK signaling proteins was respectively detected by Western blot and immunofluorescence staining, both in vitro and in vivo. After the Na-Mt treatment of HCEC-B4G12 cells for 12 h and 24 h, both ERK1/2 and p38 showed significant activation,  and JNK was not activated until 24 h after treatment   as demonstrated by their increased phosphorylation (Fig. 7A & B), whereas the total levels of these proteins had barely changed. For example, after applying Na-Mt at a concentration as low as 1.56 μg/mL to HCEC-B4G12 cells for 12 h, the level of p38 phosphorylation was about 1.3-fold that of the control group (Fig. 7C). After 24 h, the level of JNK phosphorylation in the 6.25 μg/mL Na-Mt treatment group was 1.8-fold that of the control group (Fig. 7D).

We also further verified that Mt activated MAPK signaling pathway in vivo by immunofluorescence staining of corneal tissues. Similarly, the results showed that JNK, ERK1/2, and p38 were each significantly activated in the corneal tissues of rats exposed to Na-Mt and C-H-Na-Mt for 7 days, as demonstrated by their increased phosphorylation, while the total levels of all three proteins were virtually unchanged (Fig. 7E–J). For example, compared with the vehicle control, phosphorylation of p38 in corneal tissues increased by ca. twofold at 7 days after exposure to 2 mg/mL of either Na-Mt or C-H-Na-Mt (Fig. 7K & L). Likewise, for both Mts, the phosphorylation of p38 in corneal tissues increased by ca. threefold at 7 days post-exposure to 10 mg/mL of either (Fig. 7K & L). The phosphorylation of ERK1/2 and JNK showed similar trends, but JNK was activated at a relatively weak level, this being consistent with the results of in vitro experiments.

### NAC pretreatment regulates the MAPK signaling pathway

**Fig. 8:**
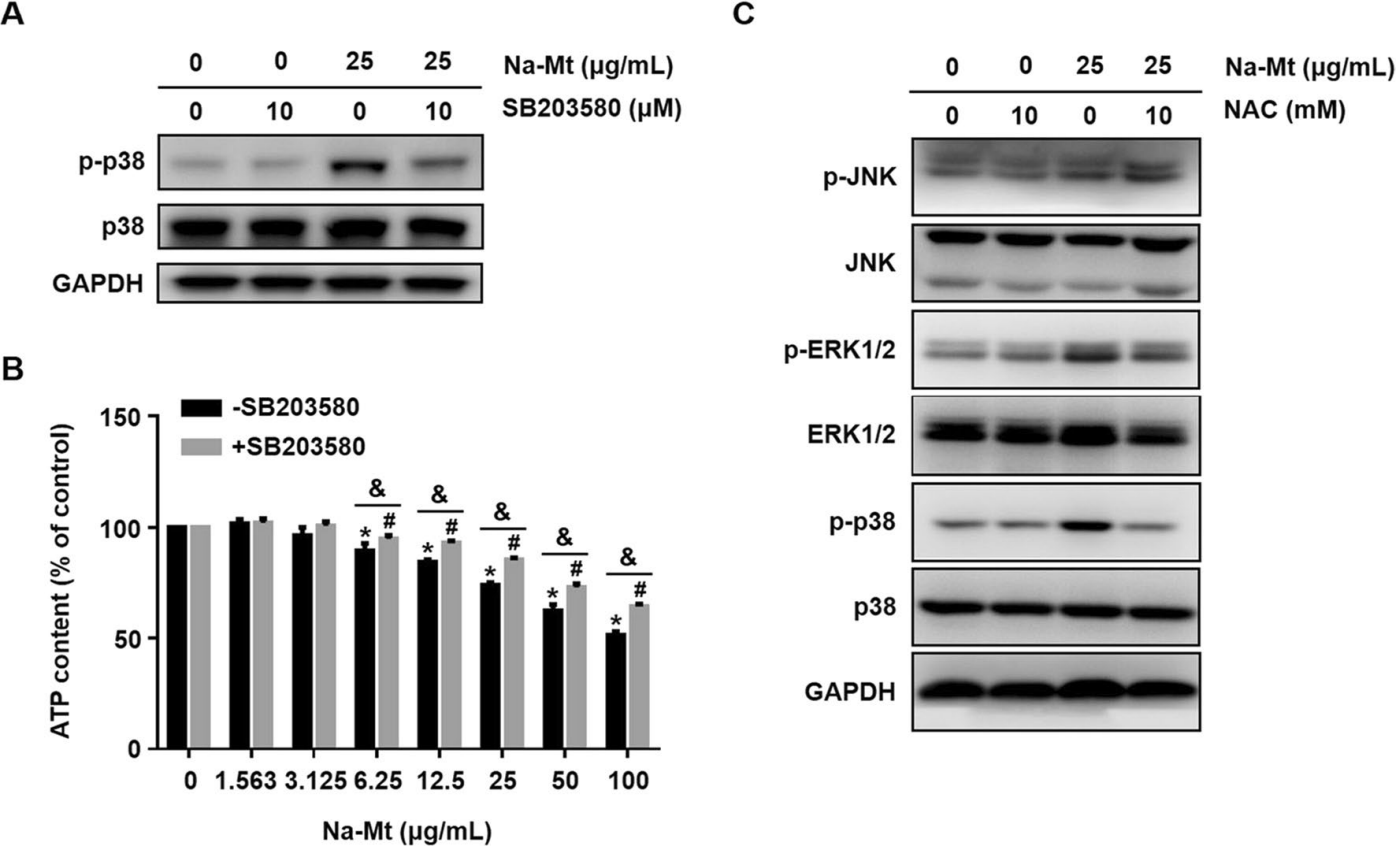
Effects of NAC pretreatment on the MAPK signaling pathway. **A**, **B** HCEC-B4G12 cells were pretreated with 10 μM SB203580 (p38 inhibitor) for 2 h prior to a treatment with Na-Mt for additional 24 h. The expression levels of p38 and p-p38 were investigated by western blot analysis (**A**). ATP content was measured by the CellTiter-Lum Plus Luminescent Cell Viability Assay (**B**). **C** Representative western bands of JNK, p-JNK, ERK1/2, p-ERK1/2, p38, and p-p38 in HCEC-B4G12. HCEC-B4G12 cells were pretreated with 10 mM NAC for 1 h before exposing them to 25 μg/mL Na-Mt for an additional 24 h. GAPDH is the loading control (**A**, **C**). Data points are the mean ± SD from three independent experiments, with three parallel samples per concentration in each experiment. *p < 0.05 compared with the control lacking the SB203580 pretreatment. ^***#***^*p* < 0.05 compared with the control receiving the SB203580 pretreatment. ^*&*^*p* < 0.05 between treatments with and without SB203580 pretreatment at the same concentration of Na-Mt

To investigate the biological significance of MAPK phosphorylation for Mt-induced cytotoxicity, HCEC-B4G12 cells were pretreated for 2 h with specific inhibitors targeting p38 (SB203580), ERK1/2 (U0126), and JNK (SP600125), followed by exposure to 25 μg/mL Na-Mt for 24 h. The decrease in ATP content induced by Na-Mt was partially rescued by pretreatment with 10 μM SB203580 (Fig. 8B), whereas pretreatment with SP600125 or U0126 did not significantly affect the ATP content vis-à-vis the Na-Mt treatment-only group (Additional file 1: Fig. S4B & D). The western blot further confirmed the efficiency of SB203580, SP600125, and U0126 at inhibiting p38 (Fig. 8A), JNK (Additional file 1: Fig. S4A), and ERK1/2 (Additional file 1: Fig. S4C), respectively, with no effect on the total protein level of each. These results revealed that, although these three MAPK signaling proteins are phosphorylated, only p38 contributes to Na-Mt-induced cytotoxicity.

As mentioned above, Na-Mt treatment induced the ROS production of HCEC-B4G12 cells. We next investigated whether ROS generation influenced functioning of the MAPK signaling pathway. For this, HCEC-B4G12 cells were pretreated with 10 mM NAC for 1 h to remove ROS, and then exposed to Na-Mt for 24 h. The western blot results suggested that the NAC pretreatment partly reversed the p38 phosphorylation status, but it did not alter phosphorylation of either JNK or ERK1/2 (Fig. 8C). This implies that p38 activation following Na-Mt treatment was triggered, at least in part, by the generation of ROS.

## Discussion

Exploring the effects of Mt on the eye is important for assessing human health risks and potential ocular applications. Although Mt is widely used, their toxic potential has not been well studied so far, especially its corneal toxicity, which is still unclear. Here, a comprehensive study of five types of Mt (Na-Mt, H-Na-Mt, C-H-Na-Mt, Ca-Mt, and MMt) and their corneal toxicity in vitro and in vivo was performed, and the underlying mechanism of Mt-induced corneal toxicity experimentally explored in detail.

Previous studies reported that Mt exerts toxic effects on kidney cells [15], hepatoma cells [27], bronchial epithelial cells [28], and mice lung tissue [29]. To our best knowledge, this is the first study to show that corneal toxicity can arise from exposure to different types of Mt. Notably, both the unmodified Mt and modified Mt have been used as drug delivery carriers in studies of ocular diseases; however, the toxicity of these Mt types in the eye has gone overlooked. For example, bromonidine, an α2-adrenergic receptor agonist, is inserted into the interlamellar space of the Na-Mt lattice to increase the preocular surface retention time of the drug and to reduce intraocular pressure in glaucoma patients [23]. In addition to pristine Na-Mt, studies have shown that some organically modified Mts, such as H-Na-Mt [19, 24, 25] and C-H-Na-Mt [26], can also deliver eye drops to delay drug release and increase drug bioavailability for use in glaucoma therapy. In addition to medical applications, Mt, such as Ca-Mt [41] and MMt [13], has also been widely studied for application in the field of environmental governance and food packaging. The eye is a highly special and sensitive organ that can be exposed to various substances due to direct exposure to the environment. As a drug carrier, Mt may have side effects on the eye, and Mt dispersed in the environment also has a risk of damage to the eye. Therefore, the exploration of ocular toxicity of Mt can not only provide basis for the clinical applications of Mt, but also provide support for the risk assessment of Mt in the industrial application. Accordingly, in our study, Na-Mt, H-Na-Mt, C-H-Na-Mt, Ca-Mt, and MMt were chosen for their corneal toxicity assessment, and human HCEC-B4G12 corneal cells were used in the in vitro assays.

Since the safety profile of Mt may be changed by their organic modification [29, 42], we first characterized the different types of Mt to compare the differences between their physicochemical properties and to verify the successful Mt modifications (Fig. 1). The SEM and TEM enabled us to analyze the surface morphology of Na-Mt, H-Na-Mt, C-H-Na-Mt, Ca-Mt, and MMt. Overall, Ca-Mt appears to have less cracking and smoother surfaces relative to Na-Mt. The many spherical nanoparticles appearing on the surface of Mt after its Fe_3_O_4_ treatment confirmed the successful preparation of MMt. Acid washing of Na-Mt using H_2_SO_4_ increased wrinkles to the edge of the thin layer; subsequent introduction of chitosan into H-Na-Mt significantly reduced the presence of nanosheets. Furthermore, the successful preparation of H-Na-Mt and C-H-Na-Mt was verified by XRD and EDS mapping. After successfully obtaining the two pristine Mts (i.e., Na-Mt and Ca-Mt) and the three organically modified Mts (i.e., H-Na-Mt, C-H-Na-Mt, and MMt), we were in a sound position to investigate their toxicity to cornea cells, both in vitro and in vivo.

To confirm whether Mt and its derivatives are capable of corneal toxicity, we performed extensive cytotoxicity tests using HCEC-B4G12 cells. ATP content, cell viability and LDH leakage are widely used indicators in cytotoxicity assays [30, 39]. All five tested Mt types induced different magnitudes of cytotoxicity in the HCEC-B4G12 cells. With a greater Mt concentration and longer exposure time, ATP content and cell viability of HCEC-B4G12 cells gradually decreased, while LDH leakage gradually increased, suggesting that enhanced Mt cytotoxicity was time- and concentration dependent (Fig. 2). Except for MMt, among the Mts, the other four types exhibited high cytotoxicity, of which Na-Mt was clearly the most toxic; hence, H-Na-Mt, C-H-Na-Mt, and MMt as modified based on Na-Mt decreased the cytotoxicity of HCEC-B4G12 cells to varying degrees, respectively. This finding is similar to that reported in a recent study [29], in which modifying the Mt with quaternary ammonium compounds reduced its pulmonary toxicity to exposed mice. By contrast, other research reported that the organic modification of Mt could increase its hazard potential. For example, Cloisite 30B, an organically modified Mt nanoclay, induced higher toxic effects relative to the original nanoclay in human lung cells [28]. In addition, Sharma et al. [43] explored the genotoxicity of natural clay mineral Mt (Cloisite®Na^+^) and organo-modified Mt (Cloisite®30B) in Caco-2 cell models, and their results showed that compared with non-genotoxic Cloisite®Na^+^, the organo-modifier (quaternary ammonium compound) incorporated in Cloisite®30B may be an important reason of genotoxicity. Connolly et al. [42] prepared two organoclays, by introducing hexadecyl trimethyl ammonium bromide and octadecyl trimethyl ammonium chloride into bentonite, respectively; both increased cytotoxicity in different cell models, including HaCaT skin cells, C3A liver cells, and J774.1 macrophage-like cells. Similarly, another study reported that surface modified nanoclays could reduce cellular inflammation and genotoxicity in in vitro models of alveolar epithelial cells (A549) and activated THP-1 macrophages (THP-1a) [44]. From these findings, it can be found that different organic modification methods can either reduce the cytotoxicity of Mt or increase the cytotoxicity of Mt, which may be related to the compounds grafted on Mt.

When cells are treated with nanomaterials and inorganic materials with large particle sizes, most of these materials are taken up by cells and they can even enter key organelles such as mitochondria, thus producing toxic effects in these cells [45–47]. Here we employed TEM to visualize the distribution of different types of Mt in cells and the negative intracellular changes induced by their treatment with various Mts (Fig. 3). TEM images confirm that all five types of Mt are taken up by HCEC-B4G12 cells within 24 h and localized within the cytoplasm, which implies the underlying mechanism of cytotoxicity induced by Mts may be attributed to the overproduction of ROS caused by Mt entry into cells, which further leads to cell damage. An in vitro study using J774.1 macrophages demonstrated that cells can uptake organoclays, which are then encased in multiple intracellular vesicles in the cytoplasm [42]. Similarly, in our study, intracellular vesicles containing different types of Mt were clearly detected in the cytoplasm of HCEC-B4G12 cells and some of these cells were undoubtedly in a state of Mt phagocytosis. Notably, a large amount of Na-Mt is attached to the cell membrane but not so in the cytoplasm, which could explain why Na-Mt is more toxic. Moreover, several studies have shown that certain nanomaterials, including SiO_2_ and CeO_2_ nanoparticles (NPs), can alter the morphology of cells [30, 37, 48]. Thus, we further investigated whether the cytotoxicity and the cellular uptake of Na-Mt, H-Na-Mt, C-H-Na-Mt, Ca-Mt, and MMt are capable of affecting the morphology of HCEC-B4G12 cells. As seen in Fig. 4, HCEC-B4G12 cells without an Mt treatment were evenly distributed, having a regular shape and smooth surface, whereas the density of cells exposed to the five types of Mt decreased in a time- and dose-dependent manner, and their cell membrane was damaged and the cell contour was ambiguous. This result is consistent with another study that found Fe^3+^-Mt damaged the cell membrane and changed the morphology of A549 cells [49]. That Na-Mt caused the most severe morphological changes, whereas MMt could only elicit significant morphological changes at a high concentration, is consistent with their cytotoxicity testing results (Fig. 2).

As mentioned above, we selected Na-Mt (pristine Mt) and C-H-Na-Mt (organically modified Mt) as representatives for the follow-up in-depth toxicity tests, because of their distinctive toxic effects and known use in ocular applications. Guided by published experimental methods [48, 50], we then examined the in vivo injuries and apoptosis to the cornea of rats exposed to Na-Mt or C-H-Na-Mt exposure through 1-week repeated exposure experiments (Fig. 5). Consistent with in vitro cytotoxicity, all these latter results showed concentration-dependent corneal toxicity of Na-Mt and C-H-Na-Mt, being higher from Na-Mt.

Oxidative stress is generally the most commonly studied factor after toxic effects in toxicology studies. The excessive increase in ROS generation due to mitochondrial damage disrupts the balance between oxidative pressure and the natural antioxidant defense system, resulting in oxidative stress [51, 52]. Previous studies have found that oxidative stress may be an important toxicological mechanism responsible for the adverse health effects of inorganic nanomaterials and air pollution particles [30, 45, 53–55]. Work by Maisanaba et al. [27] demonstrated that Mt (Cloisite®Na^+^) at relatively high concentrations can induce oxidative stress in HepG2 cells that is accompanied by increased ROS production. An earlier study obtained similar results, in which Mt (Cloisite Na^+^®) induced a significant increase in ROS production in HepG2 cells; however, ROS production did not occur in cells treated with organoclay (Cloisite 93A®) [56]. The excessive production of ROS, as an upstream event, can trigger oxidative stress that ultimately leads to cell death [39]. Our experimental data prove, for the first time, whether in vivo or in vitro, that exposure to Mt with different properties drives ROS overproduction in corneal cells (Fig. 6). Crucially, in vitro, ROS production induced by Mt in HCEC-B4G12 cells was time- and concentration-dependent; likewise, ROS production in corneal tissues exposed to Mt in vivo was also concentration-dependent. It is worth noting that, in the in vitro experiments, the trend in ROS production induced by the parent Na-Mt is of decreasing after attaining its peak value, whereas that induced by modified C-H-Na-Mt is one of continual increase. A reason for this disparity could be that the Na-Mt treatment quickly leads to the disappearance of mitochondria in cells or even the death of many cells, so that ROS can no longer be produced, in contrast to the C-H-Na-Mt treatment under the same conditions, which causes far less damage to cells. Furthermore, the inhibition of ROS significantly lessened the rate of cellular ATP depletion induced by the different types of Mt (Fig. 6), suggesting that ROS accumulation is the upstream event that triggers cytotoxicity, this being consistent with our previous findings [30, 37, 39]. Additionally, we found that Mt activated the MAPK signaling pathway both in vitro and in vivo (Fig. 7). MAPK signaling entails the involvement of p38 MAPK, JNK, and ERK, which participate in various physiological and pathological processes [39, 57]. Interestingly, ROS is known to mediate the activation of p38, JNK, and ERK1/2 signaling molecules in the MAPK signaling pathway [58]. In our study, pretreatment with NAC, an antioxidant that scours for ROS, attenuated the Mt-induced increase of p-p38, revealing that p-p38 activation was in fact ROS-mediated (Fig. 8C). The use of a p38 MAPK inhibitor also provided evidence that p38 is paramount for Mt-induced cell death (Fig. 8A&B). However, ERK1/2 and JNK were phosphorylated under Mt exposure (Fig. 7), but the NAC treatment did not affect their activation (Fig. 8C), which suggests these processes may not be directly related to ROS. Meanwhile, pretreatment with ERK1/2 and JNK inhibitor did change the ATP content compared with treatment Mt alone (Additional file 1: Fig. S4). We should emphasize that ROS inhibition only partially alleviated the cytotoxicity caused by Mt. The further causes of Mt-induced toxicity remain to be investigated.

## Conclusions

This study is the first to have investigated the corneal toxicity of different types of Mt in vitro and in vivo and its underlying mechanism. For five types of Mt, namely Na-Mt, H-Na-Mt, C-H-Na-Mt, Ca-Mt, and MMt, their induced cytotoxicity in HCEC-B4G12 cells was inferred from a lowered ATP content and diminished cell viability, increased LDH leakage and ROS generation, and the degree of altered cell morphology. At the same time, our results also show that the toxicity of Na-Mt without any modification exceeds that of Ca-Mt or any of the modified Mts (H-Na-Mt, C-H-Na-Mt, and MMt), with that of MMt being the least. Western blot analysis enabled us to uncover the potential toxicity mechanism, whereby the MAPK signaling pathway plays a unique role in regulating Na-Mt induced cytotoxicity. Consistent with the in vitro results, Na-Mt and the modified C-H-Na-Mt also induced toxicity of the rat ocular surface, caused ROS overproduction, and activated the p38, ERK1/2, and JNK proteins in MAPK signaling pathway in vivo. Notably, overproduction of ROS appears figure prominently in corneal toxicity induced by montmorillonite distinguished by its different properties. Our study provides a timely and fruitful research direction and novel insights into Mt-induced corneal toxicity and its underlying mechanisms, which could be used in the ocular administration of Mt and other similar material involving the eye. For a better understanding of which signal pathways play a crucial role in Mt-induced corneal toxicity, additional experimental studies are now needed.

## Supplementary Information

**Additional file 1: Fig. S1.** XPS spectra of N 1s (A), Si 2p (B), and O1s (C) spectra of H-Na-Mt and C-H-Na-Mt. **Fig. S2.** FTIR analysis of Mt-based materials. **Fig. S3.** Aggregation of five types of Mt incubated in FBS with different concentrations for 48h. (A) Light microscope images of Mt. (B) Hydrodynamic diameter of Mt. **Fig. S4.** JNK and ERK1/2 inhibitors do not alter Na-Mt-induced cytotoxicity. HCEC-B4G12 cells were pretreated with 10 µM SP600125 (JNK inhibitor) or U0126 (ERK1/2 inhibitor) for 2 h prior to a 24-h treatment with Na-Mt. The levels of p-JNK and JNK were detected by Western blot (A), as did the levels of p-ERK1/2 and ERK1/2 (C). ATP content was measured by CellTiter-Lum Plus Luminescent Cell Viability Assay (B and D). Data points are the mean ± SD from three independent experiments, with three parallel samples per concentration in each experiment. * and # indicate p < 0.05 compared to the vehicle control without or with pretreatment of the inhibitor.

## Data Availability

The raw data required to reproduce these findings are available within the article and its supplementary information files. The processed data required to reproduce these findings are available from the corresponding author upon reasonable request.
