# Supplementary material for: ROS generation and p-38 activation contribute to montmorillonite-induced corneal toxicity in vitro and in vivo
Source: Part Fibre Toxicol. 2023 Mar 10;20:8. doi: 10.1186/s12989-023-00519-9 (PMC9999669; doi:10.1186/s12989-023-00519-9)
Supplement: Supplementary file 1 — Additional file 1: Fig. S1. XPS spectra of N 1s (A), Si 2p (B), and O1s (C) spectra of H-Na-Mt and C-H-Na-Mt. Fig. S2. FTIR analysis of Mt-based materials. Fig. S3. Aggregation of five types of Mt incubated in FBS with different concentrations for 48h. (A) Light microscope images of Mt. (B) Hydrodynamic diameter of Mt. Fig. S4. JNK and ERK1/2 inhibitors do not alter Na-Mt-induced cytotoxicity. HCEC-B4G12 cells were pretreated with 10 µM SP600125 (JNK inhibitor) or U0126 (ERK1/2 inhibitor) for 2 h prior to a 24-h treatment with Na-Mt. The levels of p-JNK and JNK were detected by Western blot (A), as did the levels of p-ERK1/2 and ERK1/2 (C). ATP content was measured by CellTiter-Lum Plus Luminescent Cell Viability Assay (B and D). Data points are the mean ± SD from three independent experiments, with three parallel samples per concentration in each experiment. * and # indicate p < 0.05 compared to the vehicle control without or with pretreatment of the inhibitor. [file 12989_2023_519_MOESM1_ESM.docx]

Supporting Information

**ROS generation and p-38 activation contribute to montmorillonite-induced corneal toxicity *in vitro* and *in vivo***

Jia Liu^a1^, Shubin Yang^b1^, Laien Zhao^a1^, Feng Jiang^c^, Jianchao Sun^d^, Shengjun Peng^a^, Ruikang Zhao^a^, Yanmei Huang^a^, Xiaoxuan Fu^a^, Rongrui Luo^a^, Yu Jiang^a^, Zelin Li^a^, Nan Wang^a^, Tengzheng Fang^a^, and Zhuhong Zhang*

^a^School of Pharmacy, Key Laboratory of Molecular Pharmacology and Drug Evaluation (Yantai University), Ministry of Education, Collaborative Innovation Center of Advanced Drug Delivery System and Biotech Drugs in Universities of Shandong, Yantai University, Yantai 264005, P.R. China

^b^School of Chemistry and Chemical Engineering, Yantai University, Yantai 264005, P.R. China

^c^Department of Ophthalmology, Tianjin Medical University General Hospital, Tianjin 300052, P.R. China

^d^School of Environment and Material Engineering, Yantai University, Yantai 264005, People’s Republic of China

* Correspondence: [zhzhang0608@ytu.edu.cn](mailto:zhzhang0608@ytu.edu.cn)

^1^ These authors contributed equally to this work


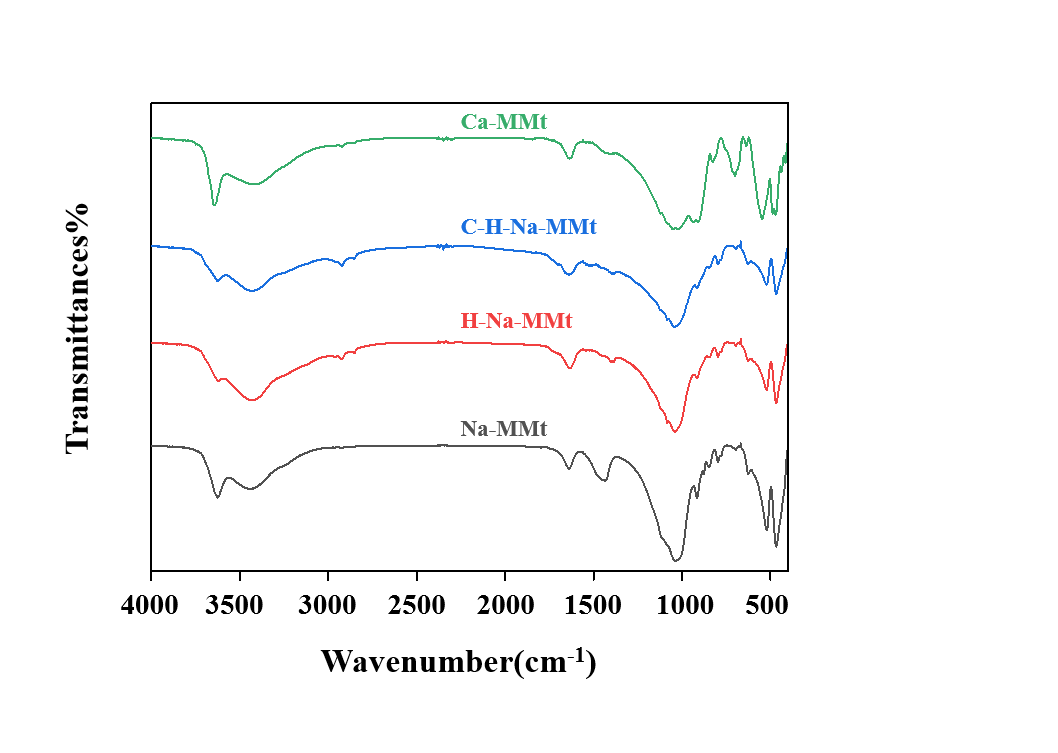

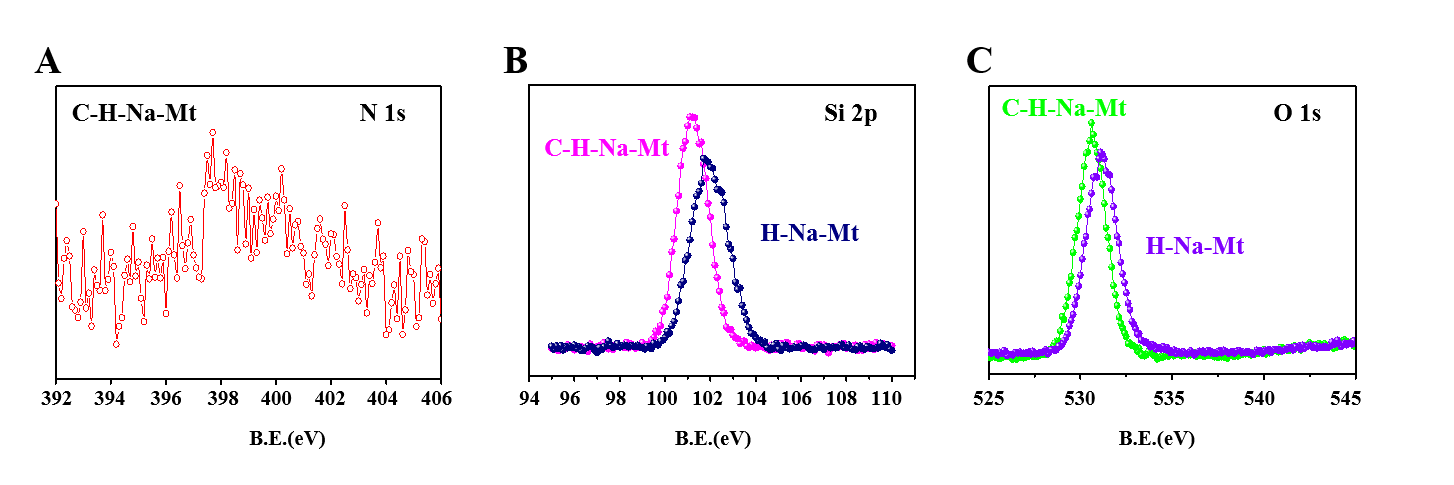
**Fig. S1.** XPS spectra of N 1s (A), Si 2p (B), and O1s (C) spectra of H-Na-Mt and C-H-Na-Mt.

**Fig. S2.** FTIR analysis of Mt-based materials.


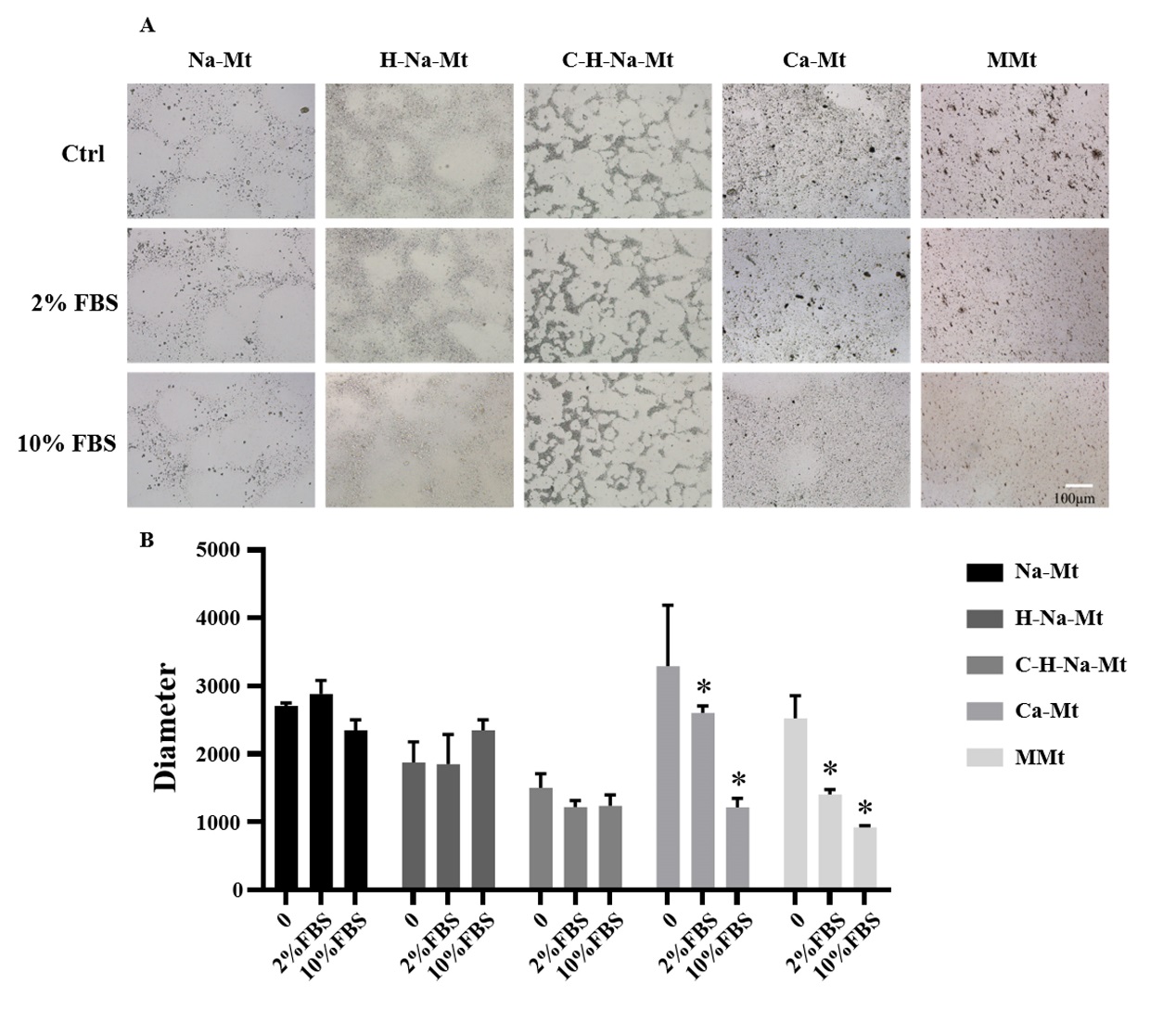
 **Fig. S3.** Aggregation of five types of Mt incubated in FBS with different concentrations for 48h. (A) Light microscope images of Mt. (B) Hydrodynamic diameter of Mt.


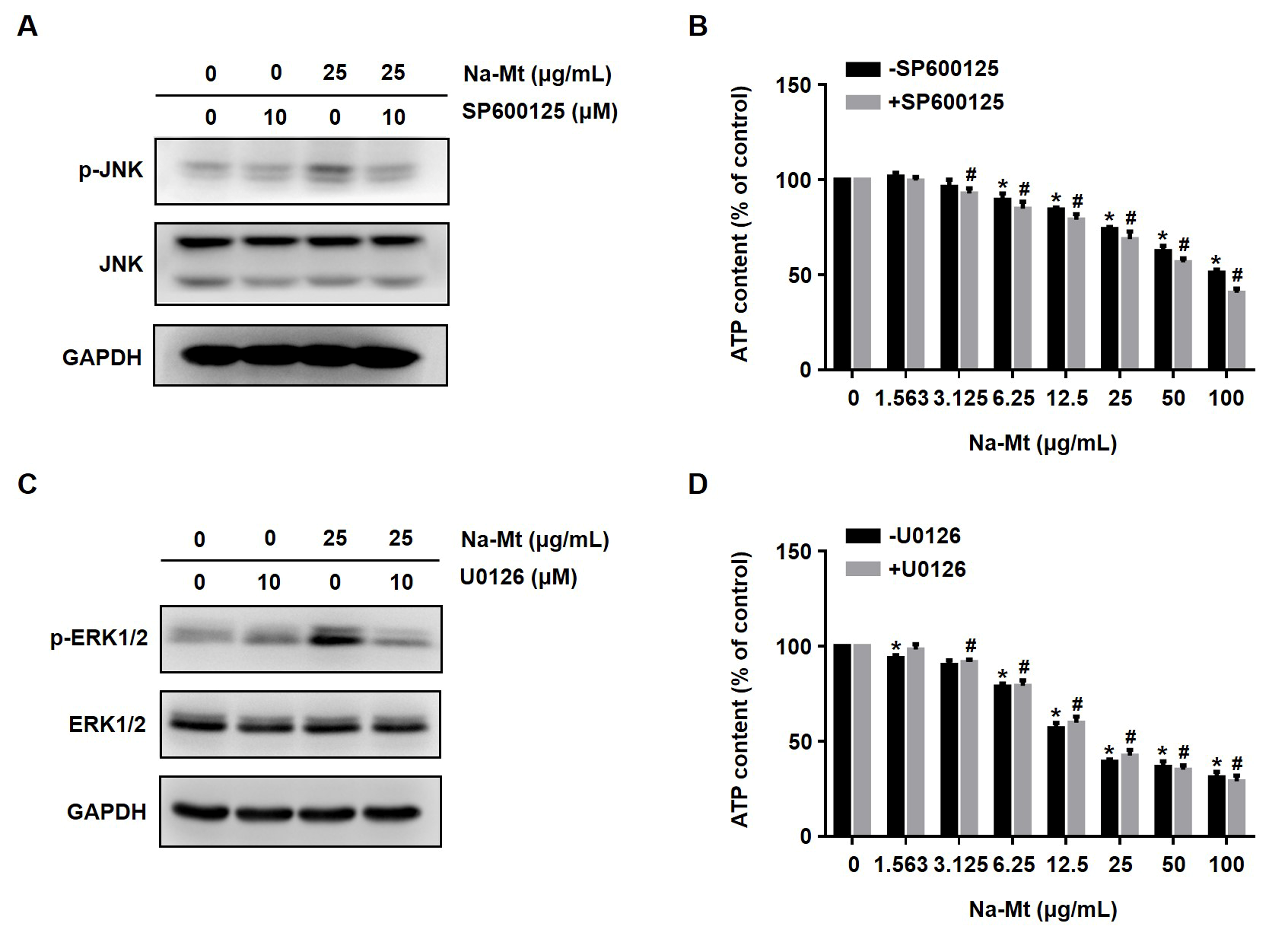


**Fig. S4.** JNK and ERK1/2 inhibitors do not alter Na-Mt-induced cytotoxicity. HCEC-B4G12 cells were pretreated with 10 µM SP600125 (JNK inhibitor) or U0126 (ERK1/2 inhibitor) for 2 h prior to a 24-h treatment with Na-Mt. The levels of p-JNK and JNK were detected by Western blot (A), as did the levels of p-ERK1/2 and ERK1/2 (C). ATP content was measured by CellTiter-Lum Plus Luminescent Cell Viability Assay (B and D). Data points are the mean ± SD from three independent experiments, with three parallel samples per concentration in each experiment. * and # indicate p < 0.05 compared to the vehicle control without or with pretreatment of the inhibitor.
